# Chromosome-scale assembly with a phased sex-determining region resolves features of early Z and W chromosome differentiation in a wild octoploid strawberry

**DOI:** 10.1093/g3journal/jkac139

**Published:** 2022-06-06

**Authors:** Caroline M S Cauret, Sebastian M E Mortimer, Marcelina C Roberti, Tia-Lynn Ashman, Aaron Liston

**Affiliations:** Department of Botany and Plant Pathology, Oregon State University, Corvallis, OR 97331, USA; Department of Botany and Plant Pathology, Oregon State University, Corvallis, OR 97331, USA; Department of Botany and Plant Pathology, Oregon State University, Corvallis, OR 97331, USA; Department of Biological Sciences, University of Pittsburgh, Pittsburgh, PA 15260, USA; Department of Botany and Plant Pathology, Oregon State University, Corvallis, OR 97331, USA

**Keywords:** sex chromosomes, whole-genome assembly, polyploid, strawberry

## Abstract

When sex chromosomes stop recombining, they start to accumulate differences. The sex-limited chromosome (Y or W) especially is expected to degenerate via the loss of nucleotide sequence and the accumulation of repetitive sequences. However, how early signs of degeneration can be detected in a new sex chromosome is still unclear. The sex-determining region of the octoploid strawberries is young, small, and dynamic. Using PacBio HiFi reads, we obtained a chromosome-scale assembly of a female (ZW) *Fragaria chiloensis* plant carrying the youngest and largest of the known sex-determining region on the W in strawberries. We fully characterized the previously incomplete sex-determining region, confirming its gene content, genomic location, and evolutionary history. Resolution of gaps in the previous characterization of the sex-determining region added 10 kb of sequence including a noncanonical long terminal repeat-retrotransposon; whereas the Z sequence revealed a *Harbinger* transposable element adjoining the sex-determining region insertion site. Limited genetic differentiation of the sex chromosomes coupled with structural variation may indicate an early stage of W degeneration. The sex chromosomes have a similar percentage of repeats but differ in their repeat distribution. Differences in the pattern of repeats (transposable element polymorphism) apparently precede sex chromosome differentiation, thus potentially contributing to recombination cessation as opposed to being a consequence of it.

## Introduction

Sex chromosomes originate with the establishment of a trigger for sex determination on a pair of autosomes. Recombination is often reduced in the vicinity of the sex-determining region (SDR), which can cause the sex chromosomes to accumulate differences (see Wright *et al.* 2016 for a review). These differences can remain limited (homomorphic) or be substantial and visible at the karyotypic level (heteromorphic). There is a continuum from homomorphic to heteromorphic sex chromosomes. Examples from the 2 extremes include a single missense mutation associated with sex in the pufferfish with no recombination suppression detected on the sex chromosomes (Kamiya *et al.* 2012) while some rodents have lost their degenerated Y chromosome altogether (Sutou *et al.* 2001).

Theoretically, one might expect the level of divergence to correlate with the time since the establishment of the sex chromosomes and recombination suppression, which is consistent with the evolution of mammalian sex chromosomes (Graves 2006). However, especially in plants, this often is not the case with multiple examples showing that the size of the nonrecombining region does not reflect the age of the SDR (reviewed in Renner and Müller 2021). A striking example is the rapid evolution of heteromorphy seen in *Silene* (Bernasconi *et al.* 2009). Recombination cessation between sex chromosomes has been associated with chromosomal rearrangements such as inversion, seen for example in papaya (Wang, Na, *et al.* 2012). An X-autosome fusion is responsible for the sex chromosomes cytotypes, which seem to be a driver of population divergence in *Rumex hastatulus* (Beaudry *et al.* 2020). However, the differential evolution (homomorphy and heteromorphy) of homologous sex chromosomes in spinach is not linked to a large-scale chromosomal rearrangement (Fujito *et al.* 2015). To understand these different evolutionary trajectories, it is useful to examine newly established sex chromosomes.

When sex chromosomes stop recombining, similar genomic hallmarks arise regardless of the system. How fast those hallmarks arise and spread, however, is still unclear. Sex chromosomes can be characterized in terms of (**1**) accumulation of mutations, (**2**) loss and gain of sex chromosome-specific regions, and (**3**) repeat sequence abundance. (**1**) After recombination ceases, the sex chromosomes start to accumulate sequence differences with the sex-limited chromosome (Y or W) accumulating deleterious mutations due to multiple evolutionary processes linked with a decrease in the efficacy of selection (Charlesworth and Charlesworth 2000). (**2**) This can cause the Y or W chromosome to degenerate, i.e. lose genes and other functional sequences. For example, almost half of the functional genes on the Y chromosome of *Silene latifolia* have been lost (Papadopulos *et al.* 2015). The sex-limited chromosomes can also acquire novel regions, for example transposable elements (TEs; described below), or the region responsible for sex determination. (**3**) Repeat sequences tend to accumulate in the nonrecombining region of sex chromosomes (Charlesworth 2013). In *Coccinia grandis*, the accumulation of repeats has led to the increase in size of the Y chromosome (Sousa *et al.* 2016). TEs have been suggested as drivers of the early step of sex chromosome differentiation and might contribute to recombination cessation (Chalopin *et al.* 2015; Almeida *et al.* 2020). TEs appear to also have an important role in sex chromosomes turnover (Faber-Hammond *et al.* 2015; Yang *et al.* 2021), as well as a role in heterochromatin formation (Zhou *et al.* 2013) and dosage compensation (Ellison and Bachtrog 2013).

Different methods are used to characterize the nonrecombining region of a sex chromosome depending on the genomic hallmarks described above. Assuming the availability of a haploid reference genome, shotgun reads from both sex chromosomes (Z and W or X and Y) will map to the same region resulting in an increase in heterozygosity in the heterogametic sex due to the accumulation of mutations (**1**). *F*_ST_ (statistics of genetic differentiation), difference in single nucleotide polymorphism (SNP) density or diversity between females and males is typically used to characterize homomorphic sex chromosomes with low level of divergence. When the sequence divergence between the sex chromosomes becomes high (**2, 3**), reads from one chromosome will not map against the sequence of the other chromosome potentially resulting in a difference in read coverage between males and females (Palmer *et al.* 2019). Repeats (**3**) can be identified de novo by for example self-alignment of a genome (Bao and Eddy 2002), using abundant k-mers, substrings of length k, from an assembly (Price *et al.* 2005) or directly from short reads (Koch *et al.* 2014) or by relying on the comparison with an existing repeat library (Smit 2004).

Because of these genomic processes, sex chromosomes are difficult to assemble with short-read sequencing (Muyle *et al.* 2017); with undifferentiated and highly differentiated sex chromosomes having different challenges. Undifferentiated sex chromosomes in particular have a low level of nucleotide divergence that makes it difficult to phase using SNPs during the assembly process. High-quality long-read sequencing can resolve the above challenges from both sex chromosomes types (heteromorphic and homomorphic). An example of the potential of this recent technology is the individual assembly of the homomorphic sex chromosomes of an eel which includes a gap free Y chromosome using a combination of PacBio HiFi and chromosome conformation capture (HiC) sequencing (Xue *et al.* 2021).

**Fig. 1.**
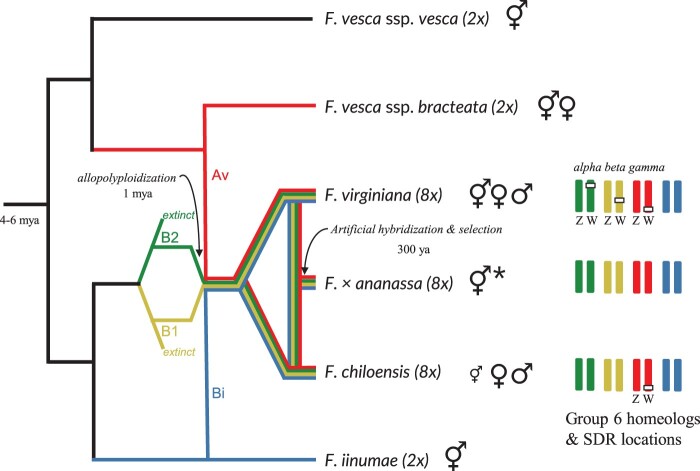
Genome and sex determination evolution in *Fragaria*. The scenario for genome evolution is simplified and adapted from Tennessen *et al.* (2014, 2018), Liston *et al.* (2020), and Session and Rokhsar (2020). The number of polyploidization events is still debated (Tennessen *et al.* 2014; Session and Rokhsar 2020) and is depicted here as a single event for simplification. The colors of the branches correspond to the octoploid subgenomes and their diploid progenitors: Av (red), Bi (blue), B1 (yellow), and B2 (green). ⚥♂♀ illustrates the presence of hermaphrodite, male, and female individuals, respectively, in a species. The original male sterility trait was selected against through selective breeding (Liston *et al.* 2014) resulting in hermaphroditism in *F.* × *ananassa* which is represented by ⚥ *. *Fragaria chiloensis* is predominantly dioecious with some occurrence of hermaphrodite individuals (represented by a small **⚥  **) including subspecies *sandwichensis* which is only hermaphrodite (Staudt 1999). The group 6 homeologous chromosomes and location of the SDR are shown on the right for the octoploid clades.

*Fragaria chiloensis* and *Fragaria* *virginiana* are octoploid species that possess a homomorphic ZW sex-determining system, i.e. the female is the heterogametic sex (Spigler *et al.* 2008; Goldberg *et al.* 2010; Tennessen *et al.* 2016, 2018), and their SDR is homologous (Tennessen *et al.* 2018, Fig. 1). These 2 species are the wild ancestors of the hybrid garden strawberry *Fragaria* × *ananassa* (Liston *et al.* 2014). Phylogenetic analysis suggests that the octoploid clade (*F. chiloensis* and *F. virginiana*) shared a common ancestor approximately 1 Mya (Dillenberger *et al.* 2018). Assuming their SDR is no older than the species, their SDR represents the youngest genetically characterized plant sex chromosome (Feng, Sanderson, *et al.* 2020). It is possible that the alpha octoploid SDR, containing only the female-specific gene *RPP0W*, was already present in the presumed extinct (Liston and Ashman 2021) diploid progenitor of the subgenome B2 (Fig. 1). However, hermaphroditism is the ancestral condition in *Fragaria*, and dioecy (females and males) is restricted to the polyploid species (Njuguna *et al.* 2013). While the closest living relative of one diploid progenitor of the octoploids, *Fragaria vesca* subsp. *bracteata*, is gynodioecious (females and hermaphrodites), its male sterility is mechanistically different, involving both cytoplasmic and nuclear genes (Ashman *et al.* 2015). The octoploid species arose by allopolyploidization and to refer to the chromosomes (or loci on those chromosomes) that are from different progenitor species and “combined” in our octoploid *F. chiloensis*, we will use the terms “homeolog” and “homeologous” throughout the text (Fig. 1).

The octoploid *Fragaria* SDR was initially linkage-mapped to 3 different homeologs of chromosome 6 in *F. chiloensis* and *F. virginiana* (Spigler *et al.* 2008; Goldberg *et al.* 2010; Tennessen *et al.* 2016). These loci were determined to represent a single homologous SDR (Tennessen *et al.* 2018) using low-coverage Illumina sequencing (2–7× per chromosome) of 31 female and 29 male plants representing the taxonomic diversity and North American geographic range of *F. chiloensis* and *F. virginiana*. Short sequences (31-mers) shared by 29 of the 31 females, and absent in all males, were used to identify a 966 bp intron-less *ribosomal protein P0* (*RPP0W*) as a candidate locus for sex determination. This locus and 1,700 bp of flanking sequence were assembled from paired-end 150 bp sequences containing the shared 31-mers. Phylogenetic analysis identified 3 clades (alpha, beta, and gamma), corresponding to the 3 linkage-mapped chromosomal locations of sex determination (Fig. 1). All plants of *F. chiloensis* belong to the gamma-clade, and identification of *F. chiloensis* female-specific 31-mers allowed assembly of a 28 kb female-specific haplotype, hypothesized to be the SDR. This haplotype contains nested sequences corresponding to alpha-clade and beta-clade female-specific 31-mers, leading to the “move-lock grow” hypothesis that the SDR has sequentially translocated by a cut-and-paste mechanism, increasing the length of the female-specific, nonrecombining region with each translocation (Tennessen *et al.* 2018).

There were 3 important limitations of this previous study: (1) An octoploid reference genome (Edger *et al.* 2019) was not available at the time, and thus the physical position of the SDR in each clade was inferred from the diploid, hermaphroditic, *F. vesca* genome. (2) The 150-bp paired-end reads were too short to allow a contiguous assembly of the SDR haplotype, resulting in 2 gaps. (3) The Z chromosome is present in both females (ZW) and males (ZZ), and thus Z-specific sequence could not be characterized, and potential sequence loss (degeneration) of the W chromosome could not be evaluated.

Here, we focus on the sex chromosomes of *F. chiloensis* which contains the version of the SDR with the greatest length and the youngest sex chromosomes (most recent translocation). To fully characterize the SDR and address the above limitations we produced a chromosome-scale assembly, which combined with previously published whole-genome sequencing (WGS) of wild individuals, allowed us to obtain the complete SDR sequence and confirm its gene content and location; use genetic differentiation (*F*_ST_) and read coverage to determine the boundaries of recombination suppression at the SDR; evaluate the extent, if any, of W degeneration (sequence loss and repeat accumulation); and test the translocation hypothesis using phylogenetic analysis.

## Methods

We used a combination of WGS HiFi long reads and short reads to further characterize the sex chromosomes of *F. chiloensis*. The resulting partially phased haplotype-resolved assembly as well as population genomics analysis (*F*_ST_, coverage) allowed us to confirm and more precisely define the limit of the nonrecombining region on the sex chromosomes, as well as to identify potential W chromosome degradation. We use the terms “Z-haplotype,” “W-haplotype,” and “haplotig” (see *Genome assembly*, *annotation*, and *evaluation*) to refer to the phased portion of the sex chromosomes in our female individual GP33, “W-specific,” “Z-specific,” and “Z-linked” to refer to the nonrecombining region of the sex chromosomes of *F. chiloensis* at the population level, and the SDR for the hemizygous region contained between the female-specific (W) *glucan endo-1,3 beta-glucosidase* and *inactive purple acid phosphatase 16* genes (Tennessen *et al.* 2018).

The 28 haploid chromosomes of the octoploid genomes can be grouped into subgenomes of 7 chromosomes each representing 4 diploid progenitors (Supplementary Table 1). While the diploid ancestry of 2 subgenomes is considered resolved, the progenitors of the remaining 2 subgenomes remain controversial (see Supplementary Methods for more information). We follow the subgenome nomenclature of Tennessen *et al.* (2014) and subgenome designations of Session and Rokhsar (2020). Thus, in the octoploid, chromosomes are numbered 1–7 and the 4 subgenomes are labeled as Av, Bi, B1, and B2 (Fig. 1).

### PacBio HiFi long-read preparation and sequencing

High molecular weight DNA was obtained from leaf tissue of a single female individual possessing the gamma-clade SDR collected from Honeyman State Park, OR, USA (GP33, USDA PI 612489; Goldberg *et al.* 2010) using the QIAGEN Genomic-tip kit (20-G size). The extraction and the HiFi sequencing library preparation were done at the Center for Qualitative Life Sciences (CQLS, Oregon State University). The library was sequenced on 2 Sequel II SMRT cells at the University of Oregon.

### Genome assembly, annotation, and evaluation

To construct a chromosome-scale genome of the gamma-SDR female *F. chiloensis* GP33, we used PacBio HiFi reads to obtain a haplotype-resolved assembly (hifiasm-0.13, Cheng *et al.* 2021). Hifiasm produces an unphased primary assembly, which contains blocks from each haplotype linked together, and an alternate assembly containing haplotigs which are haplotype-specific contigs. The primary assembly was then scaffolded using linkage mapping information as well as the *F.* × *ananassa* “Camarosa” octoploid genome (Edger *et al.* 2019) using ALLMAPS (Tang *et al.* 2015), and RagTag v1.1.1 (Alonge *et al.* 2019). The linkage maps were obtained with Onemap 2.1.3 (Margarido *et al.* 2007) using previously published target capture sequences (Tennessen *et al.* 2016). Information from the linkage maps as well as comparison to diploid and octoploid references and read coverage was used for manual curation. Pseudomolecules were oriented based on the diploid *F. vesca* (v4.0.a1) genome (Edger *et al.* 2018). Annotations from *Fragaria × ananassa* “Camarosa” v. 1.0.a2 (Liu *et al.* 2021) were transferred to our assembly using Liftoff (Shumate and Salzberg 2021). Repeats were identified and masked by RepeatMasker version 4.1.0 (http://www.repeatmasker.org) using the repeat library from *F. × ananassa* “Camarosa.” *Fragaria* genome assemblies, annotations, and the repeat library were obtained from the Genome Database for Rosaceae (Jung *et al.* 2019).

To evaluate the accuracy of the assembly, minimap2 v.2.19 (Li 2018) was used to align it against the above diploid and octoploid references. We also reconstructed sex-specific linkage maps using the final genome assembly and target capture short-read data from 44 progeny of a cross from the same individual (GP33) used for the HiFi sequencing (Tennessen *et al.* 2016). More information about the genome assembly process and quality evaluation is provided in the Supplementary Methods.

To evaluate genome completeness, we used BUSCO version 5.0.0 (Manni *et al.* 2021) with the v10 OrthoDB release (www.orthodb.org) to assess the presence and duplication level of 2,326 conserved single-copy orthologs in the *F. chiloensis* GP33 genome assembly. For comparison, we also analyzed the *F.* × *ananassa* “Camarosa” genome assembly (Edger *et al.* 2019). Telomere sequences were identified from the RepeatMasker output, using the 14 possible variations (strand and starting nucleotide) of the canonical plant telomere repeat, 5’-CCCTAAA-3′.

### Identification and confirmation of the Z and W sequences

To recover the Z and W homologous regions of the SDR, we used our haplotype-resolved hifiasm assembly. The SDR region was identified with BLAST (Altschul *et al.* 1990) using the published sequence (Tennessen *et al.* 2018) on the primary and alternate assemblies. After obtaining the SDR region in the alternate assembly, the whole haplotig was BLAST searched to both (primary and alternate) assemblies. The Z homologous region of the SDR was found in the primary assembly (as expected with a haplotype-resolved assembly). The entire haplotig that contains the SDR represents a W sequence inherited from the maternal plant (W-haplotype), consequently, its homologous region on the primary assembly represents a Z sequence inherited from the paternal plant (Z-haplotype). These Z-haplotype and W-haplotype sequences are the only phased regions in our assembly. These 2 haplotype sequences were then aligned using nucmer v.3.1 (Kurtz *et al.* 2004).

The W-haplotig and its homologous Z-haplotype sequence were confirmed at the population level by a coverage analysis. We used short reads of wild unrelated *F. chiloensis* plants from the west coast of the United States and Canada (Tennessen *et al.* 2018; Hardigan *et al.* 2020) consisting of 12 females (carrying the W SDR) and 12 males/hermaphrodites (without the W SDR; Supplementary Table 2). Adapters and low-quality regions (Q < 10) were removed using bbduk (bbmap version 01.02.2018, Bushnell 2014) and reads shorter than 50 bp were discarded. Remaining reads were mapped to our assembly using bwa mem v.0.7.17-r1188 (Li 2013) and duplicate alignments were marked with samtools fixmate/markdup v. 1.10 (Li *et al.* 2009). The read depths at each position of the W-haplotype and Z-haplotype were obtained via samtools depth. Ratio of the mean females/mean males [log2(female/male)] on a nonoverlapping window of 10 kb was computed after correcting the depth of each individual by the mean depth of a representative autosome (Fchil3-B1). For autosomal and pseudoautosomal regions a value of 0 (no difference between coverage in males and females), for W-specific region a value much larger than 0 (beyond the 95% confidence interval obtained by resampling *a* representative autosome, Fchil3-B1, 1,000 times), and a value of -1 for Z-specific region are expected.

### Sex chromosome differentiation

Except for the haplotig containing the SDR genes and its homologous region, we do not know which region of the assembled sex chromosome corresponds to the Z or W haplotypes in our focal plant GP33. To infer the sex chromosome differentiation on the rest of the sex chromosome (a mosaic pseudomolecule containing both Z and W parental sequences), we looked at the read coverage as described above. In homomorphic sex chromosomes, we expect a low level of differentiation between the Z and W chromosomes and thus a limited difference in term of coverage as both reads from the Z and the W chromosomes will map to the same region. However, nucleotide differences between the sex chromosomes can accumulate fast even in young nonrecombining regions. Thus, to define the limits of the nonrecombining region, we used our final haploid assembly (which does not contain the W specific sequence with the SDR) and estimated the divergence between unrelated wild males and females (13 of each sex, Supplementary Table 2) using *F*_ST_. Specifically, we expect a higher *F*_ST_ between females and males in the nonrecombining region of the sex chromosomes. A combination of samtools v.1.10 and bcftools v. 1.9 (Danecek *et al.* 2021) was used to call and filter genotypes (see Supplementary Methods). *F*_ST_ values were calculated on a nonoverlapping window of 10 kb using the Weir and Cockerham estimator as implemented in vcftools v.0.1.17 (weighted *F*_ST_).

TEs have a putative role in the SDR movement in strawberry (Tennessen *et al.* 2018) and the willow family (Yang *et al.* 2021) and they are known to rapidly accumulate even in young sex chromosomes (Chalopin *et al.* 2015; Almeida *et al.* 2020). In addition, it remains unclear if TEs can play a role in the cessation of recombination of the sex chromosome, see for example Furman *et al.* (2020) for a review. For these reasons, we examined the distribution of TEs across the genome using the results from RepeatMasker (see *Genome assembly, annotation, and evaluation*). The density of TEs per 10-kb window was obtained using bedtools v2.30.0 (Quinlan 2014).

### Phylogenetic analysis of the SDR (W-specific) genes and the W haplotig

Phylogenetic analysis of the 1.4 Mbp GP33 W haplotig and chromosome 6 homeologs was conducted to evaluate rates of sequence evolution between the sex chromosomes and autosomes. Homeologous sequences were obtained for 4 primary contigs and 3 alternate haplotigs from the GP33 assembly and 5 diploid *Fragaria* species (Supplementary Table 3). These 12 sequences were aligned with Mauve (Darling *et al.* 2004) identifying 67 conserved blocks (Supplementary Table 4). Partitioned maximum likelihood analysis and concordance analysis were conducted with IQ-TREE2 (Minh *et al.* 2020). Homeologous exchange (HE) is well-documented among the subgenomes of octoploid *Fragaria* (Tennessen *et al.* 2014; Edger *et al.* 2019; Liston and Ashman 2021). To examine whether HE occurs within or near the SDR, discordance among blocks was used to identify regions of HE. Additional details on the sequence alignment and phylogenetic analyses are provided in the Supplementary Methods. The above 12 sequences plus the homologous region from the *F.* × *ananassa* “Camarosa” genome were also aligned with MAFFT v7.487 (Katoh and Standley 2013) to examine a Z-specific region identified in the coverage analysis. The W haplotig and homologous Z-haplotype sequence were self-aligned with LASTZ v.1.02.00 (Harris 2007) to search for large (1 kb) palindromes, following Zhou *et al.* (2020).

To test the hypothesis of SDR translocation in the context of the octoploid genome, phylogenetic analysis was conducted for the 5 annotated genes in the SDR (Supplementary Table 5). Coding sequences were extracted from the GP33 W haplotig, and candidate orthologs were obtained using BLAT (Kent 2002) to search predicted coding sequences from the *F.* × *ananassa* “Camarosa” reannotation (Liu *et al.* 2021), the *F. vesca* Hawaii 4 reannotation (Li *et al.* 2019), and the *F. chiloensis* GP33 Liftoff annotation. For each SDR gene, the resulting sequences were aligned with MAFFT v7.487 (Katoh and Standley 2013). Best-fitting models of nucleotide sequence evolution and maximum likelihood trees with 1,000 bootstrap replicates were estimated with IQ-TREE2 (Minh *et al.* 2020). Synteny of putative SDR orthologs in Camarosa and GP33 was evaluated using MCScanX (Wang, Tang, *et al.* 2012) with the chromosome 6 homeologs.

## Results and discussion

### A chromosome-scale genome assembly to study sex chromosome evolution

**Fig. 2.**
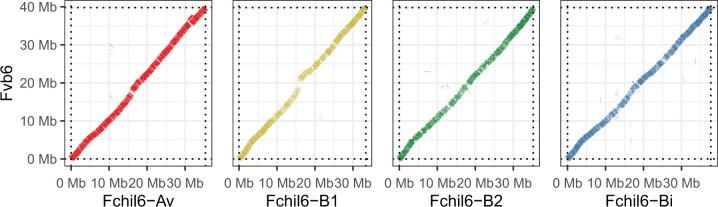
Dotplot of a minimap2 alignment between *F. chiloensis* group 6 homeologous chromosomes (Fchil6-Av, Fchil6-B1, Fchil6-B2, and Fchil6-Bi) and *F. vesca* Fvb6.

We generated a total of 6M long reads corresponding to 64 Gbases of unique molecular yield. The primary hifiasm assembly represents 859.4 Mb across 682 nuclear contigs (179 additional contigs were identified as mitochondrial and chloroplast origins) with an initial N50 of 10.8 Mb. After further scaffolding with linkage maps, and diploid and octoploid reference genomes, as well as manual refinement, we obtained a final assembly consisting of 28 main pseudomolecules corresponding to the 28 octoploid chromosomes (Supplementary Table 1). The final assembly represents about 98% of the expected genome size. The alignments against the diploid *F. vesca* and the octoploid *F.* × *ananassa* “Camarosa” showed a high degree of synteny for most chromosomes suggesting a high accuracy of our assembly (Fig. 2, Supplementary Figs. 1 and 2). Large-scale chromosome rearrangements in the “Camarosa” reference relative to the *F. vesca* genome have been previously reported (Hardigan *et al.* 2020) and are apparent when compared to our assembly (Supplementary Fig. 2). Most of these are errors and were subsequently corrected in the newly released *F.* × *ananassa* “Royal Royce” reference genome (Hardigan *et al.* 2021). Whole-genome alignments against *F. vesca* (Supplementary Fig. 1), “Camarosa” (Supplementary Fig. 2), and “Royal Royce” (Supplementary Fig. 3) suggest that using “Camarosa” in one of our scaffolding steps did not adversely impact our assembly. Genetic linkage mapping provided further evidence that the overall quality of the GP33 genome assembly is high except for one chromosome, Fchil4-B2 (Supplementary Table 6). Fchil4-Bi contains a sequence from Fchil4-B2 while the rest of the chromosome appears accurate. The alignment against “Royal Royce” shows 2 chromosomes with differences, located at the beginning of Fchil1-Bi and the end of Fchil3-B2 (Supplementary Fig. 3). Further investigation is needed to resolve whether these are misassemblies or true rearrangements.

To evaluate genome quality, we examined the presence of conserved genes and telomeres. BUSCO analysis found that 99.1% of conserved single-copy orthologs are present in the *F. chiloensis* GP33 genome assembly (Supplementary Table 7), very similar to the percentage for *F.* × *ananassa* “Camarosa.” The 2 genomes share 16 missing single-copy orthologs, an additional gene is uniquely missing in both. When examined by subgenome (Supplementary Table 7), a much lower number of conserved single-copy orthologs are observed in the B subgenomes (81.6–86.8%) than the Av subgenome (95.2%), consistent with other evidence for its dominant status in the octoploid genome (Edger *et al.* 2019). The Camarosa genome has a much higher percentage of duplicated conserved single-copy orthologs, especially for the Av subgenome (11.1% vs 2.5%) with the GP33 genome being closer to diploid *F. vesca* (2.1%). Likewise, Camarosa has 7 chromosomes with 4× or more duplicated genes than the diploids, while GP33 had only one such chromosome (Supplementary Table 8). This inflated number of duplicates may result from the incorporation of divergent haplotypes into the Camarosa assembly. A BUSCO analysis of the octoploid “Royal Royce” genome obtained very similar results to the GP33 genome reported here (Hardigan *et al.* 2021).

Putative telomeric sequences (>750 bp, mean = 1,102 bp) were found at the 5’ and/or 3’ ends in 25 of the 28 GP33 chromosome pseudomolecules, and 7 were assembled telomere-to-telomere (Supplementary Table 9). Two additional pseudomolecules are potentially telomere-to-telomere, but the putative telomeric sequences are located 270 kb or 2.4 Mbp from one chromosome end. Eleven chromosomes had a short (mean = 115 bp) interstitial telomere-like sequence (Supplementary Table 9). Overall, 60% of expected telomeres were assembled, indicative of a high-quality assembly.

### The W haplotig confirms the SDR location and gene content but reveals a larger size

**Fig. 3.**
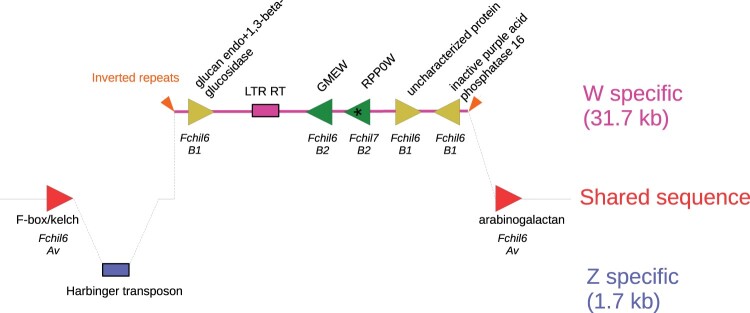
Gene and TEs on the W-specific *F. chiloensis* SDR and its homologous Z sequence. The W-specific region corresponds to the pink line, the Z to the blue area. Triangles represent genes, their direction shows the reading frame. Genes nomenclature follows (30). Gene colors correspond to the subgenome they originated from: Av (red), B1 (yellow), and B2 (green). An asterisk in *RPP0W* highlights its origin by retrotransposition from a gene on Fchil7-B2 while the other SDR genes resulted from sequential translocation from Fchil6-B2 and Fchil6-B1, respectively. The chromosomes from which the genes originated from are labeled in italics under the genes. Boxes represent TEs, and the orange arrows denote inverted repeats flanking the SDR insertion. The diagram is not to scale.

To identify the SDR in our *F. chiloensis* genome assembly, we used BLAST with the previously identified gamma-SDR sequence (Tennessen *et al.* 2018). The entire SDR insertion of 31,455 bp flanked by two 29 bp inverted repeats (Fig. 3, Supplementary Fig. 4) was found within a 1.4 Mb haplotig (in the alternate assembly). The nucleotide sequence also confirms the structure, orientation and order of the 5 W specific genes previously annotated: *glucan endo-1,3 beta-glucosidase*, *GDP-mannose-3′,5′-epimerase* (*GMEW*), *ribosomal protein P0* (*RPP0W*), uncharacterized protein, and *inactive purple acid phosphatase 16* (Fig. 3, Supplementary Fig. 4). The W chromosome SDR insertion is flanked by 2 predicted genes, *F-box kelch* and *arabinogalactan*. Each has a copy on the Z chromosome Fchil_6-Av, consistent with the expected Av subgenome location of the gamma-SDR. The W *F-box kelch* differs from the Z copy (and *F. vesca* and Camarosa) by a single 3 bp in-frame insertion of leucine. The W *arabinogalactan* differs from the Z copy by a single nonsynonymous change (glycine → aspartic acid) which is apparently derived on the Z and in Camarosa.

The intron-less *RPP0W* is a candidate gene for sex determination (Tennessen *et al.* 2018). Although not reported by Tennessen *et al.* (2018), we found it possesses 2 features that are a hallmark of retrotransposition: a 16 bp poly-adenine sequence located 99 bp downstream of its stop codon and a pair of 13 bp direct repeats located adjacent to the poly-adenine and 152 bp upstream of the start codon (Supplementary Fig. 4). While common in mammals, retrotransposition is relatively rare in plant genomes, comprising only 1–-5% of duplicated “pseudogenes” in 5 examined genomes (Mascagni *et al.* 2021). Such retrotransposed intron-less genes are historically called processed pseudogenes, on the assumption that they will not produce proteins due to the absence of regulatory sequences. However, an increasing number are now recognized as producing functional proteins, DNAs or RNAs, and it has been proposed that calling them retrogenes is more appropriate (Cheetham *et al.* 2020). In fact, *Lethe* is a well-studied long noncoding RNA (lncRNA) in mice derived from a retrotransposed ribosomal protein; it has an important regulatory role in modulating inflammatory responses (Rapicavoli *et al.* 2013). We speculate that *RPP0W* may also be an lncRNA. If *RPP0W* is an lncRNA, it would represent another example of sex determination in plants via noncoding RNA, as known in persimmon (Akagi *et al.* 2014, 2016) and the Salicaceae (willow and poplar family, Yang *et al.* 2021). This hypothetical function of *RPP0W* still requires experimental confirmation.

Even though the assembled sex chromosome (Fchil6-Av) is a mosaic of both the maternal W and paternal Z sequences, the haplotype-resolved assembler (hifiasm) allowed us to obtain the homologous SDR region of the Z-chromosome which was previously unknown. In our final haploid assembly, the SDR (as defined by the *F-box* and *arabinogalactan* genes) is located at ∼34.2 Mb on Fchil6-Av which is consistent with the results from linkage mapping (Tennessen *et al.* 2016).

The W- and Z- chromosomes share a conserved sequence immediately flanking the 29-bp inverted repeats that demarcate the gamma-SDR insertion, allowing the homologous location on the Z to be precisely identified (Fig. 3 and Supplementary Fig. 5). Sequence from the Z-chromosome contains a 1,703 bp insertion adjoining this location (Supplementary Fig. 5). The entire insertion corresponds to a *Harbinger* class II DNA TE with a DDE superfamily endonuclease. The *Harbinger* TE is demarcated by characteristic (Grzebelus *et al.* 2007) 14-bp terminal inverted repeats and TTA target site duplications (Supplementary Fig. 5).

The previously reported SDR assembly (Tennessen *et al.* 2018) contains 2 gaps (Supplementary Fig. 5). The gap between *GMEW* and *RPP0W* is flanked by a 594-bp region that is absent in the GP33 SDR haplotig. This region corresponds to a class II DNA TE in the *hAT* superfamily. The gap was apparently the result of a misassembly, and there is no evidence for an *hAT* TE in the GP33 SDR.

The original SDR assembly gap between *GMEW* exons 6 and 7 contains a 10,299 bp insertion in the GP33 SDR, increasing its size by 33% (Fig. 3, Supplementary Fig. 4). A newly annotated feature is a 4,923 bp long terminal repeat (LTR) Class I retrotransposon (RT) belonging to the *Copia* superfamily. The direct repeats are 208 and 209 bp in length, and the flanking dinucleotides are noncanonical 5’-TA.TA-3’ (vs canonical 5’-TG.CA-3’). In 50 plant genomes, noncanonical motifs comprise only 1.7% of *Copia* LTR RTs (Ou and Jiang 2018). Noncanonical LTR RTs tend to be older, more likely to be flanked by nonrepetitive sequences, and closer to genes (Ou and Jiang 2018). The LTR direct repeats differ by 1 SNP and 1 indel, consistent with the recent origin of the SDR. The remaining 5,138 bp of the 10.3 kb insertion has sequence similarity to LTR RTs with ambiguous superfamily classification, but lacking the characteristics of a functional LTR. We interpret this as representing a degraded LTR RT. We did not identify any new genes in this newly gap-closed region.

The discovery of an LTR RT within the SDR and a *Harbinger* transposon on the homologous Z-sequence raises the question of their possible involvement with the translocation of the SDR (Tennessen *et al.* 2018). However, each has terminal repeat sequences that are independent of the 29 bp inverted repeats flanking the SDR. In addition, no transposase proteins are associated with the SDR flanking repeats and they are not repeated elsewhere in the GP33 genome. This suggests that the SDR translocation was mediated by a nonautonomous mechanism, as previously hypothesized (Tennessen *et al.* 2018). While many cases of transposon function have now been documented in plant genomes (Lisch 2013; Ariel and Manavella 2021), whether the SDR LTR and Z-specific *Harbinger* TE are biologically functional remains to be determined.

### Small localized region of differentiation between the Z- and W- chromosomes

**Fig. 4.**
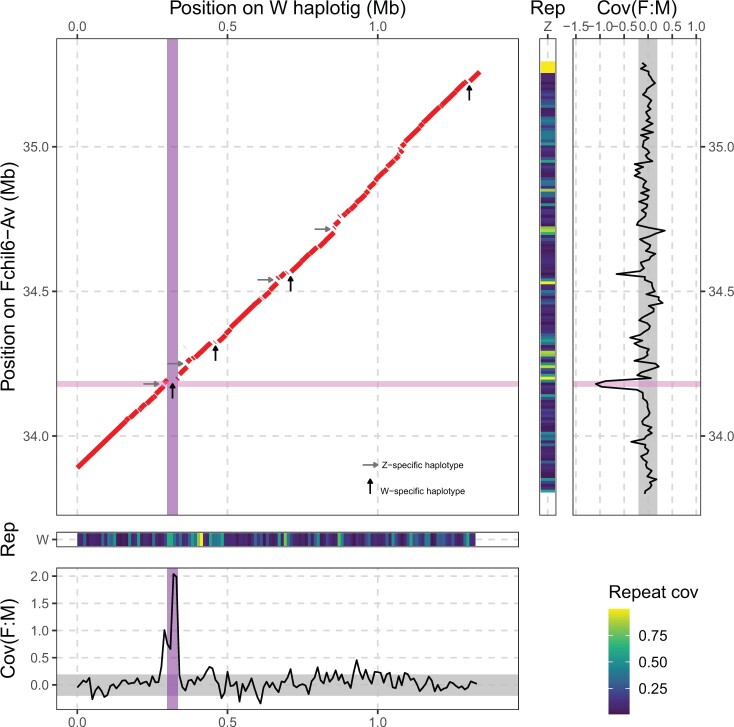
Identification and characterization of W- and Z-specific regions. Top-left: Dotplot of a nucmer alignment between the W and Z haplotype-resolved region. Specific regions of the Z-haplotype and W-haplotype sequences are highlighted by horizontal gray and vertical black arrows, respectively. Middle panels: Repeat coverage on the W (bottom) and Z (right) haplotype sequences. Bottom and far right: Coverage difference between males and females [log2(mean F) − log2(mean M)] on nonoverlapping 10-kb windows: on the W haplotig (bottom) and on the Z homologous region (right). The region highlighted in dark purple represents the SDR, in light purple a Z-specific region. Values close to 0 indicate regions shared between Z and W (PAR), −1 and > 0 potential Z- and W-specific regions, respectively.

To infer the level of divergence between the sex chromosomes, we first aligned the identified W-haplotype (haplotig containing the SDR) and its homologous Z-haplotype sequence. The 2 showed high synteny (Fig. 4) which is expected due to the young age of these sex chromosomes. The largest region of nonalignment between them corresponds to the W region containing the SDR (Fig. 4). Multiple regions appear to be Z- or W-haplotype specific in our assembly and are not restricted to the SDR and adjacent regions as illustrated, for example, by a gap in the diagonal of the dotplot (Fig. 4) corresponding to a W-haplotype-specific region toward the end of the W resolved haplotig (∼1 Mb from the SDR).

To confirm that these potential regions of high divergence are not simply the result of high haplotype divergence in our individual plant (prevalent in plant genomes; Bayer *et al.* 2021; Hämälä *et al.* 2021) but rather reflect the recombination cessation between the sex chromosomes, we used differences in sequence read coverage between geographically diverse females and males of *F. chiloensis* (Supplementary Table 2). We detected a spike in the ratio F/M coverage at 290–340 kb on the W-haplotig which includes the W-specific SDR (position 298.7–334.8 kb; dark purple in Fig. 4). A dip in the coverage difference was seen at 34.17–34.20 Mb (light purple in Fig. 4) confirming a Z-linked region. Within this region, we found a ∼10 kb sequence with no homolog on the W chromosome containing a predicted *F-box* protein-coding gene (based on BLAST search on NCBI; Supplementary Fig. 6). No other gene was found based on blast search against databases and results obtained with the Liftoff software. The other Z- and W-haplotype-specific regions in our assembly which did not show a male and female bias in the read coverage of wild individuals suggest that those regions belong to the pseudoautosomal region of the sex chromosome which still recombines despite structural variation.

**Fig. 5.**
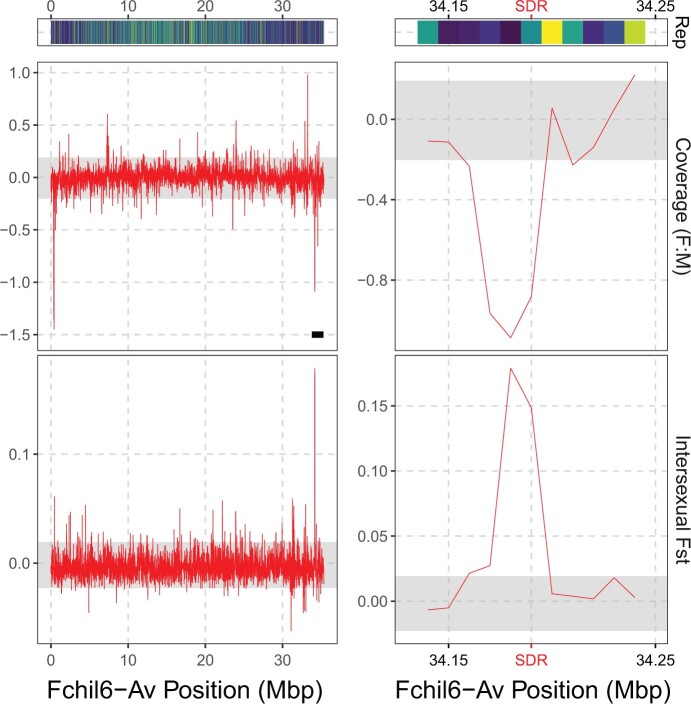
Repeat coverage, intersexual weighted F_ST_, and difference between males and females in coverage [log2(mean F) − log2(mean M)] across the sex chromosome (Fchil6-Av) in nonoverlapping 10-kb windows. The gray area represents the 95% confidence interval obtained by resampling a representative autosome (Fchil3-B1) 1,000 times. The black line on the top left panel highlights the haplotype-resolved region illustrated in Fig. 4. For the F_ST_ analysis, the reads were remapped on the reference sequence without the inclusion of the W haplotype which was included in the coverage analysis. The right panel represents a zoomed view of the region at the vicinity of the SDR insertion on the Z chromosome, defined by the F-box and arabinogalactan genes.

To confirm that the nonrecombining region of the *F. chiloensis* sex chromosomes is limited to the haplotype (Z/W) resolved region, and further characterize the border of the pseudoautosomal region, we used differences in coverage and intersexual *F*_ST_ across the whole-sex chromosome as each technique is used to detect different level of sex chromosome divergence. We saw an increase in the ratio of F/M coverage at position 33.25–33.26 Mb which is ∼1 Mbp from the SDR (Fig. 5 and Supplementary Fig. 7). The F/M difference in coverage is lower than in the region containing the known W-specific SDR. We think this spike likely reflects an artifact, since no female-specific k-mer was found in this region (see below). Interestingly, we found one additional dip (compared to Fig. 4) in the coverage at position 370–410 kb (Fig. 5), which is consistent with this region being Z-specific (coverage values around −1 with the lowest value at around −1.5). This spike is at the opposite end of the chromosome from the SDR. A fully phased haplotype-resolved assembly could determine if this represents a large Z-chromosome inversion, an assembly artifact, or another cause of low coverage in females.

At the SNP level, differentiation between females and males was detected by an elevated *F*_ST_ from positions 34.18–34.2 Mb on the sex chromosome (Fchil6-Av). This region extends beyond the location homologous to the W SDR insertion at position 34.1962 Mb. Other chromosomes showed an elevated *F*_ST_ but not of a similar magnitude (Supplementary Fig. 8). The intersexual *F*_ST_ was also examined separately for the main 2 geographic areas sampled (Oregon and California) to determine whether they differ in the extent of their nonrecombining region; however, this was inconclusive due to our small sample size (Supplementary Fig. 9, Supplementary Table 1). In a previous study (Tennessen *et al.* 2018), female-specific k-mers were identified. Eighty-nine percent of them were used to make the reference SDR sequence of *F.* *chiloensis*, but it remains unclear how far from the SDR the other k-mers occur. We found that all 651 *F. chiloensis* female-specific 31-mers map to a 46.8 kb section of the W-haplotig (from 292 to 339 kb). This result is similar from the coverage and *F*_ST_ analysis but further highlights the low level of fixed nucleotide differences between the Z and W as the first group of female-specific k-mers is separated from the second by a ∼8.2 kb region that align with a Z homologous sequence on most of its length (171 bp did not align). The concordance of the coverage, *F*_ST_ and k-mer analyses suggest that the entire nonrecombining region was fully phased in our assembly.

Sex chromosome degeneration is often described in terms of accumulation of repetitive elements such as TEs. Repeat sequences made up 19.75% of the total size of the W-haplotig vs 20.36% for the homologous Z-haplotype sequence which is inconsistent with expectations for W degeneration. *Gypsy* LTRs have accumulated on the Z-haplotype sequence while they constitute a low percentage of the W repeats (Supplementary Fig. 11). The Z- and W-haplotype-specific regions corresponded to regions rich in repeated sequences (Fig. 4). However, the whole-sex chromosome (Fchil6-Av, chimera of the parental Z—inherited from the paternal plant—and W—inherited from the maternal plant—sequences) showed a lower repeat content compared to the other chromosomes (Supplementary Fig. 12). This can be due to the subgenome origin as overall Av chromosomes show a lower repeat content than the other chromosomes. We did not observe an increase of repeats on the nonrecombining region of the sex chromosome (Fig. 4). Instead, we saw a repeat accumulation between 10 and 20 Mb (Fig. 5), which could potentially indicate a centromeric region as centromeres tend to accumulate repeats (Melters *et al.* 2013). TE polymorphism (presence on only one of the sex chromosome haplotype) contributes to structural variation between the Z- and W-haplotype sequences in our assembly even beyond the identified nonrecombining region (Fig. 4). The female W-specific SDR in *F. chiloensis* is located within a region with low recombination rates in both females and males (Tennessen *et al.* 2016), suggesting that the SDR is located in a region of pre-existing low recombination (instead of the SDR triggering recombination cessation at this location) as shown in other species (Rifkin *et al.* 2021). TE polymorphism (presence/absence) has been linked to recombination cessation in maize (Dooner and He 2008). While we did not link TE polymorphism and recombination rate in this study, we observed TE polymorphism in the pseudoautosomal region of the sex chromosomes (the phased sequences containing the SDR and its homologous region), a region known to have a low recombination rate. It would be of interest to investigate TE polymorphism on the whole-sex chromosome, on the sex chromosome homeologs, on the autosomes, and at the population levels. Since they cause the haplotypes of the sex chromosome (and potentially other chromosomes) to be diverged despite recombining, TE polymorphism may impact recombination rates and have a role in strawberry sex chromosome evolution. To test this hypothesis, fully phased assemblies of multiple individuals are needed.

The theory of sex chromosome evolution (Charlesworth *et al.* 2005) predicts that sex chromosomes accumulate differences after the emergence of a trigger for sex determination. From this, it follows that the most likely hypothesis to explain Z-specific sequences would be through W degeneration (loss of the homologous sequence). If this was the case, we would expect to find sequences homologous to the Z-specific region (position 34.17–34.20 Mb on the Z-haplotype) in the Fchil-6 homeologs as they are autosomes as well as in progenitor diploid species. Based on blast results, the Z-specific region including the *F-box* gene is homologous to a region (∼2.4 kb) on Fchil6-Bi at position ∼36.65 Mb. No homologous region was found on the other homeologous chromosomes (Fchil-6B1 and Fchil6-B2). These results are consistent with the Z-specific gene being lost on the W, and could potentially represent W degeneration. A caveat is that gene loss is also common in polyploid genomes (Otto 2007), as reflected by the absence of autosomal copies of this *F-box* gene on Fchil-6B1 and Fchil6-B2. Additional aspects of sex chromosome degeneration not explored in this paper are changes in the expression levels of sex-linked genes and the presence or not of dosage compensation.

### Sequence features and phylogenetic analysis support the translocation hypothesis

**Fig. 6.**
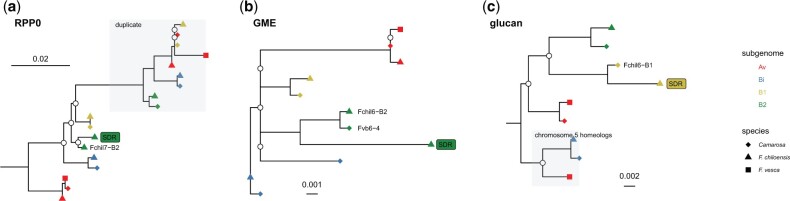
Maximum likelihood phylogenetic trees of (a) *ribosomal protein P0* (RPP0), (b) *GDP-mannose-3′,5′-epimerase* (GME), and (c) *glucan endo-1,3 beta-glucosidase* (glucan). Nodes with bootstrap support <80 are represented by white circles. The copy of the genes present in the female-specific SDR is labeled “SDR” with a rectangle filled with the subgenome color of its original autosomal copy (Av = red, B1 = yellow, B2 = green, Bi = blue). Gray areas depict genes belonging to the gene families but not homeologous to the original autosomal copy of the SDR genes. The chromosomal location is given for the sister copies of the SDR genes. Tree visualization was done in R version 4.1.0 (R Core Team 2021) using ggtree v.3.0.4 (Yu 2020). The complete phylogenies are given in the Supplementary Figs. 13–15.

Phylogenetic results for the SDR genes (Supplementary Table 5, Figs. 3 and 6) were consistent with the previously inferred translocation hypothesis (Tennessen *et al.* 2018). The first event in the establishment of the SDR was the retrotransposition of *RPP0W* from a homeolog of chromosome 7 to Fchil_6-B2. The *RPP0* gene tree supports the origin of *RPP0W* from the B2 subgenome (Fig. 6 and Supplementary Fig. 13). This raises the possibility that the retrotransposition occurred in the diploid progenitor of the octoploid. However, an origin after polyploidization cannot be ruled out. Since the B2 ancestor is likely extinct (Liston and Ashman 2021), resolving this may not be possible.

The *GMEW* gene is resolved with GP33 and Camarosa orthologs on chromosomes 6-B2 (Fig. 6 and Supplementary Fig. 14) and synteny was confirmed with MCScanX at the expected position of the alpha SDR. The GP33 ortholog is apparently derived from the former Z-chromosome at this location, consistent with the previously proposed cut-and-paste translocation (Tennessen *et al.* 2018).

Three genes are predicted to originate with the beta SDR on chromosome 6-B1, but a strongly supported sister-group relationship was only obtained for *glucan endo-1,3 beta glucosidase* (Fig. 6, Supplementary Figs. 15–17). For *purple acid phosphatase*, bootstrap support is low (Supplementary Fig. 16); nevertheless, the SDR gene has higher sequence similarity to the 6-B1 ortholog than any other octoploid homeolog. GP33 orthologs on Fchil_6-B1 were not recovered for any of these 3 genes, searching both the primary and alternate Hifiasm contigs, and the unassembled PacBio ccs reads. MCScanX analysis identified missing sequences in the GP33 assembly relative to Camarosa, between 8.3 and 10.8 Mb on Fvb6-2. Searching unassembled primary and alternate GP33 contigs for the Camarosa genes in the region identified a 1.17 Mbp primary contig (ptg000176l) that contained 87 of the 195 expected genes. This contig has a single segregating marker for linkage mapping—thus it could not be oriented and was excluded from the ALLMAPS scaffolding. After taking this contig into account, the missing genes in GP33 comprise a 1.59 Mbp gap. The lack of a W ortholog is expected according to the cut and paste translocation hypothesis, but contrary to expectations, no Z ortholog was found, either. A potential explanation is that this large region has been deleted from both the beta SDR W haplotype as well as its Z homolog. A complete assembly of this region is needed to confirm this scenario.

Phylogenetic analysis of the GP33 W-haplotig and orthologous regions of chromosome 6 from the other homeologs and diploid species found that branch length differences between the Z- and W-haplotypes were similar to other homeologs (Supplementary Fig. 15). This is consistent with the limited ZW divergence observed in the *F*_ST_ and coverage analyses. All branches had 100% bootstrap support, and most were supported by >50% of conserved blocks and nucleotide sites. These results are consistent with several other genome-scale phylogenetic analyses (Tennessen *et al.* 2014; Feng, Wang, *et al.* 2020; Liston *et al.* 2020; Session and Rokhsar 2020; Liston and Ashman 2021) and do not support the subgenome assignments published with the Camarosa genome (Edger *et al.* 2019). Some of the lower gene support is due to 3 cases of HE from the Av to the B1 and one from the Av to the Bi subgenome (Supplementary Table 10). All cases involved both haplotypes of the B genomes. HE from the Av to B subgenomes is the predominant mode of HE in the octoploid genomes (Tennessen *et al.* 2014; Edger *et al.* 2019; Liston and Ashman 2021). HE could potentially obscure or mislead the SDR translocation hypothesis, but no evidence of HE was found within 169 kb of the SDR (Supplementary Table 10).

#### General discussion and concluding remarks

We obtained a partially phased genome of a wild strawberry (*F. chiloensis*) female using HiFi PacBio long reads. Combined with a reanalysis of short-read sequencing from past research we were able to confirm without ambiguity the content and gene order of the SDR in *F. chiloensis*, and its location on Fchil6-Av. Phylogenetic analysis confirmed the previously inferred translocation history of the SDR. As expected from young sex chromosomes, we found limited signs of sex chromosome divergence, i.e. small Z- and W-specific regions, and low level of nucleotide divergence. We did not find evidence of increased repeat density on the W-resolved haplotype. However, we observed polymorphic TEs (i.e. the distribution of repeats differs) between the W- and Z-resolved haplotypes, including in the pseudo-autosomal region. TE polymorphism (observed in this study) coupled with the location of the SDR in a region of low recombination (Tennessen *et al.* 2016), is consistent with a potential role for repeat sequences in the early step of sex chromosome divergence and recombination suppression (Chalopin *et al.* 2015; Almeida *et al.* 2020).

While sex chromosome turnover has been well-documented in several animals (Cauret *et al.* 2020; Keating *et al.* 2021; Lichilín *et al.* 2021), it is still poorly known in plants. SDR translocation in the Salicaceae (poplar and willow family) has some parallels to what we have observed in octoploid *Fragaria*. Unlike willows, we found no evidence of large palindromes (Zhou *et al.* 2020) and could not associate autonomous transposons with SDR movement. Most of the characterized SDRs in Salicaceae are older and larger than observed in *Fragaria*. The most similar is *Populus alba* with ZW sex determination and a 69 kb SDR with 12 W-specific genes (Yang *et al.* 2021), compared to 31 kb and 5 W-specific genes in *F. chiloensis*. The ability to obtain high-quality chromosome-scale genome assemblies is poised to greatly accelerate the characterization of sex chromosomes (Carey *et al.* 2021), and will clarify whether the features of SDR translocation observed here are general phenomena for plants. Acquisition of haplotype-resolved assemblies, even partially phased such as in this study, will allow a better understanding of autosomal haplotype divergence (exacerbated by repeat sequences), and how this shapes the origin of sex chromosomes. Haplotype-resolved assemblies will also resolve the question of whether repeat sequences, especially TEs, are a cause or consequence of recombination suppression (Furman *et al.* 2020).

An important question that remains is what are the drivers of the SDR movement in the octoploid strawberries and other taxa with mobile SDR and more generally of sex chromosome turnover? Two nonexclusive major hypotheses explaining sex chromosome turnover, reviewed in Palmer *et al.* (2019), involve accumulation of deleterious mutations and sexual antagonistic selection. An expectation arising from the first hypothesis is a lower recombination rate in the heterogametic sex. Notably, this is not the case in *Fragaria* (Tennessen *et al.* 2016). Because different levels of sex differentiation are observed in *F. virginiania* (Ashman 2003, 2005), a species that harbors 3 different locations of the SDR, sexual antagonism seems a promising hypothesis. More data are especially needed from gene expression, as sex-biased expression is often used as a proxy for sexual conflict (Mank 2017). It is important to note that while sexual conflict can play an important role in sex chromosome evolution in animals, it seems limited in plants; in contrast, plants display a higher potential for haploid selection (Mank 2022). Due to the variability of the potential drivers of sex chromosomes in animals, for example mutation load in Ranid frogs (Jeffries *et al.* 2018) vs sexual antagonistic selection in cichlid fish (Roberts *et al.* 2009), it is important to compare animal and plant systems that possess a similar trajectory of sex chromosome evolution. For example, strawberries and salmon both have an SDR that has moved among chromosomes by translocation (Faber-Hammond *et al.* 2015; Tennessen *et al.* 2018). Testing whether the same forces result in a similar pattern of sex chromosome turnover in disparate organisms is an exciting avenue for future comparative studies.

## Data availability

The PacBio HiFi reads have been deposited in the NCBI SRA (BioProject PRJNA812950). Genome assemblies (hifiasm primary and alternate assemblies, and the final curated assembly with its annotation), sequence alignments, phylogenetic trees, and vcf files used for the *F*_ST_ calculation, along with the codes used to produce the figures are available on Zenodo (https://doi.org/10.5281/zenodo.6547728). The final curated assembly is also available on the Genome Database for Rosaceae (GDR, https://www.rosaceae.org/). Other sequencing data used in the paper are already available in the NCBI SRA (Supplementary Table 2).

Supplemental material is available at *G3* online.

## Supplementary Material

jkac139_Supplementary_Data
