# Supplementary material for: Chromosome-scale assembly with a phased sex-determining region resolves features of early Z and W chromosome differentiation in a wild octoploid strawberry
Source: G3 (Bethesda). 2022 Jun 6;12(8):jkac139. doi: 10.1093/g3journal/jkac139 (PMC9339316; doi:10.1093/g3journal/jkac139)
Supplement: jkac139_Supplementary_Data [file jkac139_supplementary_data.pdf]

Supplemental methods

Genome assembly

The PacBio HiFi long reads were initially assembled into contigs using hifiasm-0.13 (Cheng et al., 2021). The kmer size was set to 31, default values were kept for the other parameters. The primary assembly produced by hifiasm was then used as the backbone for the scaffolding step. A first scaffolding step was performed by ALLMAPS (Tang et al., 2015) using a male (markers that are heterozygous and homozygous in the paternal and maternal plant respectively), a female (homozygous/heterozygous in the paternal/maternal plant) and a combined linkage (makers used in the sex specific maps as well as markers heterozygous in both parents) maps as inputs. The genetic maps were produced with published target capture sequences (Tennessen et al., 2016). The short reads from a female, and a male *F. chiloensis*, and 44 of their offspring were mapped against the hifiasm primary assembly using the mem command of BWA version 0.7.17-r1188 (Li, 2013). Genotypes were called using the combination of samtools 1.10 and bcftools version 1.9 (Danecek et al., 2021). Six individuals were excluded from the rest of the analysis due to poor coverage using VCFtools 0.1.17 (Danecek et al., 2011). Sites with a quality score %QUAL<20, a depth (DP) <10, a genotype quality (GQ) <20, or a percentage of individuals with missing data (F_missing) > 0.1 were filtered out with bcftools. The final filtered vcf file was imported into R version 4.0.3 and converted into onemap raw format using vcfR 1.12.0 (Knaus & Grünwald, 2018) followed by onemap 2.1.3(Margarido, Souza & Garcia, 2007). Only biallelic markers with no missing data were kept for further analysis. Markers were separated depending on their segregation types and each map was made independently. A total of 4352, 2356 and 7633 were used for the female, male and combined maps respectively. Redundant markers and markers which departed from Mendelian segregation expectations were removed. The logarithm of the odds (LOD) threshold was set to 6 and the maximum recombination fraction was kept to 0.5 (default). The genetic maps were estimated using the rcd algorithm with the *make_seq* function. The linkage maps as well as comparisons with a diploid reference genome *Fragaria vesca* v4.0.a1 (Edger et al., 2018) and octoploid reference genome *Fragaria* x *ananassa* 'Camarosa' v1.0.a2 (Edger et al., 2019) highlighted errors in the initial contigs from hifiasm. RagTag (Alonge et al., 2019) was used to correct the assembly based on disagreement with the reference and low coverage, to further scaffold, and to label the pseudomolecules. The program minimap2 v.2.19 (Li, 2018) was used to map the HiFi PacBio reads (-ax map-hifi) and to align the pseudomolecules to the octoploid reference for RagTag (correct: --mm2-params '-cx asm10 -z 1000' --min-cov 4 --max-cov 50 --remove-small -q 20, scaffold: --mm2-params '-N 0 -cx asm5 -z 1000' --remove-small -q 20). Due to the persistence of errors in the assembly after ragtag, manual curation was performed for five chromosomes in which a contig was split between multiple linkage groups and between multiple chromosomes of *F. vesca*. Break points were chosen based on low coverage (HiFi reads were mapped against the genome using minimap using the map-hifi option), blast results, and the presence of "N" resulting from scaffolding. No manual correction was performed (1) if the linkage maps supported the assembly despite disagreement with the *F. vesca* reference, which was the case for Fchil2-B1; or (2) if the number of markers for a linkage group was too low to infer the quality of a contig. Errors resulting from the last ragtag scaffolding step were not curated. Annotations from *Fragaria × ananassa* 'Camarosa' v. 1.0.a2 (Liu et al., 2021) were transferred to our assembly using Liftoff (Shumate & Salzberg, 2021). Repeats were identified and masked by RepeatMasker version 4.1.0 (http://www.repeatmasker.org) using the repeat library from *F. × ananassa* 'Camarosa'. *Fragaria* genome assemblies, annotations and the repeat library were obtained from the Genome Database for Rosaceae (Jung et al., 2019). Quality of the assembly was assessed with Quast v.5.0.0

(Mikheenko et al., 2018) and by aligning the assembly onto the diploid *F. vesca* and octoploid *F. x ananassa* reference genomes with minimap2. We expect four pseudomolecules from our octoploid assembly to match each diploid chromosome, each pseudomolecule having a different level of divergence from the diploid reference. We re-built sex-specific genetic maps using the same datasets and similar methods as described above. This time all the filtered markers were used to identify the linkage groups. Sex specific maps were then obtained by separating the markers based on their segragation pattern. The command *make_seq* was used first to estimate the genetic maps as previously. If the results were deemed of poor quality based on the recombination fraction map the function *rdc* was used instead.

Genome nomenclature

The 28 haploid chromosomes can be grouped into subgenomes of 7 chromosomes each representing four diploid progenitors (Table S1). We follow the subgenome nomenclature of (Tennessen et al., 2014) and subgenome designations of (Session & Rokhsar, 2020). The four subgenomes are labelled as Av, Bi, B1 and B2. The diploid ancestry of two subgenomes is considered resolved: *F. vesca* subsp. *bracteata* is the progenitor of the Av subgenome and *F. iinumae* is the progenitor of the Bi subgenome (Rousseau-Gueutin et al., 2009; Njuguna et al., 2012; Tennessen et al., 2014; Edger et al., 2019; Session & Rokhsar, 2020; Feng et al., 2021) On the other hand, the progenitors of the B1 and B2 two subgenomes is controversial. Using phylogenetic analysis of linkage-mapped targeted sequence, no extant diploid was resolved as a sister taxon and *F. iinumae* was the closest diploid ancestor (Tennessen et al., 2014). Using the "Phylogenetic Identification of Subgenomes" approach (https://github.com/mrmckain/PhyDS), the diploids *F. nipponica* and *F. viridis* were identified as the B1 and B2 progenitors (Edger et al., 2019, 2020). Subsequent studies have not supported this disposition (Liston et al., 2020; Session & Rokhsar, 2020; Feng et al., 2021; Liston & Ashman, 2021) and agree that *F. iinumae* is the closest living ancestor of the B1 and B2 subgenomes. By examining the distribution of repetitive sequence that is over-represented in one or more subgenomes, Sessions and Rokhsar (Session & Rokhsar, 2020) provided additional support for the *F. vesca* and *F. iinumae* origin of subgenomes Av and Bi. Their analysis also suggests a novel division of the remaining chromosomes, and they named these the alpha and beta subgenomes. To avoid confusion with our SDR nomenclature, we refer to these as B1 and B2. With two exceptions (chromosomes 3 and 5) their division is consistent with Tennessen et al. (Tennessen et al., 2014). In chromosome-scale phylogenetic analyses (Liston et al., 2020; Liston & Ashman, 2021) the B1 branch is either sister to *F. iinumae* plus B2, or longer than B2 when sister taxa. This was the same criterion that Tennessen et al. used to separate these subgenomes – but due to the small amount of sequence data available, their assignment of B1 and B2 for chromosome 3 and 5 was incorrect, and is corrected in this study (Table S1).

Sex chromosome differentiation

For the coverage and Fst analysis, we originally mapped the reads of 25 males and 13 females with bwa mem (v.0.7.17-r1188) and duplicate alignments were marked with samtools fixmate/markdup (v. 1.10). For Fst a combination of samtools v.1.10 and bcftools v. 1.9 was used to call the genotypes. Sites with a low quality (<20) and with low read depth (<100) were excluded. Genotypes with low quality (<20) were set to missing. Sites with a high proportion of missing individual genotypes (>=0.3) were excluded. Due to concerns of over-representation of males in our original dataset , we excluded some from the both analyses. More specifically we kept males from the same geographical area when possible or nearby and

of similar autosomal read depth. The results were qualitatively similar (Fig. S6 and S16). To avoid infinite values, we added 0.001 to every value prior to compute the mean females to mean males ratio.

A 10 kbp Z-specific sequence contained a Liftoff (Shumate & Salzberg, 2021) annotated gene. A BLAST search of the CDS identified it as a putative Fbox protein. An homeologous copy was found on Fchil-Bi, and aligned to the Z-specific sequence with MAFFT (Katoh & Standley, 2013, Supplemental File 1).

Phylogenetic analysis of the 1.4 Mbp GP33 W haplotig and chromosome 6 homologs was conducted to evaluate rates of sequence evolution between the sex chromosomes and autosomes. Homeologous sequences were obtained for 4 primary contigs and 3 alternate haplotigs from the GP33 assembly (Table S3). An alternate haplotig was not assembled for Fchil-B2. In addition, the orthologous region of chromosome 6 was extracted from 5 diploid *Fragaria* species (Table S3). These 12 sequences were aligned with Mauve (Darling et al., 2004). Conserved blocks with 10-12 sequences were extracted and filtered to exclude gap columns present in 3 or more sequences, retaining 67 alignments over 1000bp (Table S4). Five alignments were reduced to 9 sequences, due to the gap filtering. The final alignment had 636,266 sites and 11.8% missing data. Partitioned maximum likelihood analysis and concordance analysis were conducted with IQ-TREE2 (Minh, Hahn & Lanfear, 2020), with best-fitting models of sequence evolution estimated for each conserved block. Branch support was evaluated with 1000 bootstrap replicates. Homeologous exchange (HE) is well-documented among the subgenomes of octoploid *Fragaria* (Tennessen et al., 2014; Edger et al., 2019; Liston & Ashman, 2021). To examine whether HE occurs within or near the SDR, discordance among blocks was used to identify regions of HE. To determine the location of HE in a large 30kb block, the block was split into 5 kb sections and phylogenies were estimated as described above for the single genes.

Literature cited

Alonge M, Soyk S, Ramakrishnan S, Wang X, Goodwin S, Sedlazeck FJ, Lippman ZB, Schatz MC. 2019. RaGOO: fast and accurate reference-guided scaffolding of draft genomes. *Genome Biology* 20:224. DOI: 10.1186/s13059-019-1829-6.

Cheng H, Concepcion GT, Feng X, Zhang H, Li H. 2021. Haplotype-resolved de novo assembly using phased assembly graphs with hifiasm. *Nature Methods* 18:170–175. DOI: 10.1038/s41592-020-01056-5.

Danecek P, Auton A, Abecasis G, Albers CA, Banks E, DePristo MA, Handsaker RE, Lunter G, Marth GT, Sherry ST, McVean G, Durbin R, 1000 Genomes Project Analysis Group. 2011. The variant call format and VCFtools. *Bioinformatics* 27:2156–2158. DOI: 10.1093/bioinformatics/btr330.

Danecek P, Bonfield JK, Liddle J, Marshall J, Ohan V, Pollard MO, Whitwham A, Keane T, McCarthy SA, Davies RM, Li H. 2021. Twelve years of SAMtools and BCFtools. *GigaScience* 10:giab008. DOI: 10.1093/gigascience/giab008.

Darling ACE, Mau B, Blattner FR, Perna NT. 2004. Mauve: Multiple Alignment of Conserved Genomic Sequence With Rearrangements. *Genome Research* 14:1394–1403. DOI: 10.1101/gr.2289704.

Edger PP, McKain MR, Yocca AE, Knapp SJ, Qiao Q, Zhang T. 2020. Reply to: Revisiting the origin of octoploid strawberry. *Nature Genetics* 52:5–7. DOI: 10.1038/s41588-019-0544-2.

Edger PP, Poorten TJ, VanBuren R, Hardigan MA, Colle M, McKain MR, Smith RD, Teresi SJ, Nelson ADL, Wai CM, Alger EI, Bird KA, Yocca AE, Pumplin N, Ou S, Ben-Zvi G, Brodt A, Baruch K, Swale T, Shiue L, Acharya CB, Cole GS, Mower JP, Childs KL, Jiang N, Lyons E, Freeling M, Puzey JR, Knapp SJ. 2019.

Origin and evolution of the octoploid strawberry genome. *Nature Genetics* 51:541–547. DOI: 10.1038/s41588-019-0356-4.

Edger PP, VanBuren R, Colle M, Poorten TJ, Wai CM, Niederhuth CE, Alger EI, Ou S, Acharya CB, Wang J, others. 2018. Single-molecule sequencing and optical mapping yields an improved genome of woodland strawberry (*Fragaria vesca*) with chromosome-scale contiguity. *Gigascience* 7:gix124.

Feng C, Wang J, Harris A, Folta KM, Zhao M, Kang M. 2021. Tracing the diploid ancestry of the cultivated octoploid strawberry. *Molecular Biology and Evolution* 38:478–485.

Jung S, Lee T, Cheng C-H, Buble K, Zheng P, Yu J, Humann J, Ficklin SP, Gasic K, Scott K, Frank M, Ru S, Hough H, Evans K, Peace C, Olmstead M, DeVetter LW, McFerson J, Coe M, Wegrzyn JL, Staton ME, Abbott AG, Main D. 2019. 15 years of GDR: New data and functionality in the Genome Database for Rosaceae. *Nucleic Acids Research* 47:D1137–D1145. DOI: 10.1093/nar/gky1000.

Katoh K, Standley DM. 2013. MAFFT multiple sequence alignment software version 7: improvements in performance and usability. Molecular biology and evolution 30:772–780.

Knaus BJ, Grünwald NJ. 2018. Inferring Variation in Copy Number Using High Throughput Sequencing Data in R. *Frontiers in Genetics* 9:123. DOI: 10.3389/fgene.2018.00123.

Li H. 2013. Aligning sequence reads, clone sequences and assembly contigs with BWA-MEM. *arXiv:1303.3997 [q-bio]*.

Li H. 2018. Minimap2: pairwise alignment for nucleotide sequences. *Bioinformatics* 34:3094–3100. DOI: 10.1093/bioinformatics/bty191.

Liston A, Ashman T-L. 2021. The origin and subgenome dynamics of the octoploid strawberries. In: *Acta Horticulturae*. International Society for Horticultural Science (ISHS), Leuven, Belgium, 107–118. DOI: 10.17660/ActaHortic.2021.1309.17.

Liston A, Wei N, Tennessen JA, Li J, Dong M, Ashman T-L. 2020. Revisiting the origin of octoploid strawberry. *Nature Genetics* 52:2–4. DOI: 10.1038/s41588-019-0543-3.

Liu T, Li M, Liu Z, Ai X, Li Y. 2021. Reannotation of the cultivated strawberry genome and establishment of a strawberry genome database. *Horticulture Research* 8:1–9. DOI: 10.1038/s41438-021-00476-4.

Margarido GRA, Souza AP, Garcia AAF. 2007. OneMap: software for genetic mapping in outcrossing species. *Hereditas* 144:78–79. DOI: 10.1111/j.2007.0018-0661.02000.x.

Mikheenko A, Prjibelski A, Saveliev V, Antipov D, Gurevich A. 2018. Versatile genome assembly evaluation with QUAST-LG. *Bioinformatics (Oxford, England)* 34:i142–i150. DOI: 10.1093/bioinformatics/bty266.

Minh BQ, Hahn MW, Lanfear R. 2020. New Methods to Calculate Concordance Factors for Phylogenomic Datasets. *Molecular Biology and Evolution* 37:2727–2733. DOI: 10.1093/molbev/msaa106.

Njuguna W, Liston A, Cronn R, Ashman T-L, Bassil N. 2012. Insights into phylogeny, sex function and age of *Fragaria* based on whole chloroplast genome sequencing. *Molecular Phylogenetics and Evolution* 66:17–29.

Rousseau-Gueutin M, Gaston A, Aïnouche A, Aïnouche ML, Olbricht K, Staudt G, Richard L, Denoyes-Rothan B. 2009. Tracking the evolutionary history of polyploidy in *Fragaria* L. (strawberry): New insights from phylogenetic analyses of low-copy nuclear genes. *Molecular Phylogenetics and Evolution* 51:515–530. DOI: 10.1016/j.ympev.2008.12.024.

Session A, Rokhsar D. 2020. Discovering subgenomes of octoploid strawberry with repetitive sequences. *bioRxiv*:2020.11.04.330431. DOI: 10.1101/2020.11.04.330431.

Shumate A, Salzberg SL. 2021. Liftoff: accurate mapping of gene annotations. *Bioinformatics* 37:1639–1643. DOI: 10.1093/bioinformatics/btaa1016.

Tang H, Zhang X, Miao C, Zhang J, Ming R, Schnable JC, Schnable PS, Lyons E, Lu J. 2015. ALLMAPS: robust scaffold ordering based on multiple maps. *Genome Biology* 16:3. DOI: 10.1186/s13059-014-0573-1.

Tennessen JA, Govindarajulu R, Ashman T-L, Liston A. 2014. Evolutionary origins and dynamics of octoploid strawberry subgenomes revealed by dense targeted capture linkage maps. *Genome Biology and Evolution* 6:3295–3313.

Tennessen JA, Govindarajulu R, Liston A, Ashman T-L. 2016. Homomorphic ZW chromosomes in a wild strawberry show distinctive recombination heterogeneity but a small sex-determining region. *New Phytologist* 211:1412–1423.

Supplemental results

Table S1. *Fragaria chiloensis* GP33 chromosome pseudomolecule sizes and comparison with *F.* × *ananassa* 'Camarosa'.

GP33	Length (bp)	Camarosa	Length (bp)	Camarosa / GP33	Edger (2019) chromosome number
Fchil1-Av	23959720	Fvb1-4	22887349	0.96	1
Fchil1-B1	26021663	Fvb1-1	27594200	1.06	4
Fchil1-B2	26641460	Fvb1-3	27436561	1.03	3
Fchil1-Bi	25344140	Fvb1-2	28910674	1.14	2
Fchil2-Av	29124927	Fvb2-2	24782128	0.85	5
Fchil2-B1	28786371	Fvb2-1	26582685	0.92	7
Fchil2-B2	28929757	Fvb2-3	24073015	0.83	8
Fchil2-Bi	27126548	Fvb2-4	26692599	0.98	6
Fchil3-Av	31877583	Fvb3-4	27809139	0.87	9
Fchil3-B1	31192042	Fvb3-1	32005440	1.03	12
Fchil3-B2	33321682	Fvb3-3	29626823	0.89	11
Fchil3-Bi	31374497	Fvb3-2	31459976	1.00	10
Fchil4-Av	27527683	Fvb4-3	31955388	1.16	13
Fchil4-B1	26800935	Fvb4-1	20034018	0.75	16
Fchil4-B2	24464411	Fvb4-2	25974422	1.06	15
Fchil4-Bi	37483869	Fvb4-4	26295489	0.70	14
Fchil5-Av	29583688	Fvb5-1	29826953	1.01	17
Fchil5-B1	26427873	Fvb5-4	25211045	0.95	19
Fchil5-B2	28462679	Fvb5-2	24981319	0.88	20
Fchil5-Bi	24151278	Fvb5-3	27452983	1.14	18
Fchil6-Av	35360427	Fvb6-1	36657112	1.04	21
Fchil6-B1	33373235	Fvb6-2	36124132	1.08	23
Fchil6-B2	35260406	Fvb6-4	34274104	0.97	24
Fchil6-Bi	37710691	Fvb6-3	43627644	1.16	22
Fchil7-Av	21615347	Fvb7-2	32354134	1.50	25
Fchil7-B1	22008715	Fvb7-4	23534715	1.07	28
Fchil7-B2	23120314	Fvb7-1	32186896	1.39	27
Fchil7-Bi	21038310	Fvb7-3	25137763	1.19	26
**Total**	**798090251**		**805488706**	**1.01**	

Table S2. Shotgun sequence samples of *Fragaria chiloensis* used in the Fst and coverage analyses.

ID	Sex	Location	NCBI SRA Accession	References
GP33.1* = PI612489	F	Oregon	SRR6149717	Tennessen et al 2018
EUR12.1	F	California	SRR6149676	Tennessen et al 2018
EUR13.2	F	California	SRR6149675	Tennessen et al 2018
EUR17.1	F	California	SRR6149682	Tennessen et al 2018
HM12.1	F	Oregon	SRR6149716	Tennessen et al 2018
HM16.1	F	Oregon	SRR6149718	Tennessen et al 2018
PTR14.3	F	California	SRR6149705	Tennessen et al 2018
SAL6.1	F	Oregon	SRR6149704	Tennessen et al 2018
SAL8.1	F	Oregon	SRR6149710	Tennessen et al 2018
PI551458	SDR	Washington	SRX7023237, SRX7023236	Hardigan et al. 2020
PI551468	SDR	Oregon	SRX7023229, SRX7023228, SRX7023227	Hardigan et al. 2020
PI612490	SDR	California	SRX7023253, SRX7023252, SRX7023251	Hardigan et al. 2020
PI616652	SDR	Bristish Columbia	SRX7023256, SRX7023255, SRX7023254	Hardigan et al. 2020
EUR9.1	MH	California	SRR6149679	Tennessen et al 2018
HM13.1	MH	Oregon	SRR6149719	Tennessen et al 2018
PIS3.1	MH	Oregon	SRR6149694	Tennessen et al 2018
PTR17.1	MH	California	SRR6149672	Tennessen et al 2018
SAL4.1	MH	Oregon	SRR6149673	Tennessen et al 2018
PTR19.2	H	California	SRR6149713	Tennessen et al 2018
PTR5.3	H	California	SRR6149703	Tennessen et al 2018
SAL3.1	H	Oregon	SRR6149684	Tennessen et al 2018
PIS5.1 *	MH	Oregon	SRR6149702	Tennessen et al 2018
PI551467	noSDR	Oregon	SRX7023240, SRX7023239, SRX7023238	Hardigan et al. 2020
PI551735	noSDR	Alaska	SRX7023242, SRX7023241	Hardigan et al. 2020
PI551753	noSDR	California	SRX7023234, SRX7023233, SRX7023232	Hardigan et al. 2020
PI551765	noSDR	Oregon	SRX7023249, SRX7023244, SRX7023243	Hardigan et al. 2020

* samples used for Fst but not for coverage analysis

Table S3. Sequences includes in the phylogenetic analysis of the GP33 Z and W chromosomes, autosomes, and diploid *Fragaria* species.

Sequence	Length	Haplotig or Coordinates	Source
Fchil6-Av (Z)	1470410	33890018-35360427	this publication
Fchil6-Av_atg (W)	1339445	atg000165l	this publication
Fchil6-B1	1420238	31952998-33373235	this publication
Fchil6-B1_atg	611097	atg001016l	this publication
Fchil6-B2	1016930	34243477-35260406	this publication
Fchil6-Bi	1345554	36365138-37710691	this publication
Fchil6-Bi_atg	320426	atg000414l	this publication
Fves6	1526885	38042876-39569760	*F. vesca* (Edger et al. 2018)
Fiin6	1195694	41401417-42597110	*F. iinumae* (Edger et al. 2020)
Fnil6	1580543	49098618-50679160	*F. nilgerrensis* (Feng et al. 2020)
Fnub6	1909236	38859317-40768552	*F. nubicola* (Feng et al. 2020)
Fvir6	1494318	35987002-37481319	*F. viridis* (Feng et al. 2020)

Table S4. Phylogenetic input and results for the concatenated matrix and each Mauve block in the IQ-Tree concordance analysis.

ID	Mauve block	sequences	length (bp)	informative sites	model	lnL	tree length
1	LCB_067	10	2596	59	HKY+F+G4	-5106.08	0.0928
2	LCB_080	10	25583	975	HKY+F+R3	-57685.6	0.1506
3	LCB_100	10	13227	453	HKY+F+I	-29047.9	0.1376
4	LCB_101	10	11621	635	HKY+F+G4	-29022.1	0.202
5	LCB_115	10	3039	68	HKY+F+G4	-6580.39	0.1291
6	LCB_120	10	4476	151	HKY+F+G4	-10236.5	0.1665
7	LCB_127	10	2791	72	HKY+F+G4	-5933.56	0.1301
8	LCB_128	10	6395	154	HKY+F+G4	-13167.9	0.1088
9	LCB_133	10	3265	125	HKY+F+G4	-7661.84	0.1767
10	LCB_137	10	8916	378	TPM3+F+G4	-20407.8	0.1546
11	LCB_138	10	15357	514	HKY+F+G4	-33236.1	0.1331
12	LCB_139	10	4621	175	HKY+F+G4	-10301.3	0.137
13	LCB_150	10	29783	781	TN+F+I+G4	-63259.1	0.1268
14	LCB_217	9	4710	151	TPM3+F+I	-9782.12	0.1221
15	LCB_219	10	1505	60	TPM3+F+G4	-3574.54	0.1855
16	LCB_222	9	10476	312	HKY+F+G4	-21669.9	0.1119
17	LCB_267	11	3488	148	HKY+F+I	-7717.8	0.1535
18	LCB_268	11	7534	289	HKY+F+G4	-16705.4	0.1502
19	LCB_272	11	6860	229	HKY+F+G4	-14846.9	0.1283
20	LCB_274	11	5278	216	HKY+F+R2	-11555.4	0.1492
21	LCB_277	11	20965	1198	HKY+F+R3	-44907.3	0.1533
22	LCB_300	10	13358	266	HKY+F+I	-26406.3	0.1055

23	LCB_301	10	14511	725	HKY+F+G4	-35195	0.1822
24	LCB_302	11	9400	476	K3Pu+F+R2	-21880.7	0.1744
25	LCB_312	10	1555	53	HKY+F+G4	-3533.61	0.1508
26	LCB_320	12	3112	146	HKY+F+I	-7260.9	0.1775
27	LCB_321	12	8232	289	HKY+F+G4	-17170.3	0.1096
28	LCB_328	10	4454	172	HKY+F+G4	-9982.68	0.1456
29	LCB_330	11	6983	393	HKY+F+G4	-15797.6	0.1515
30	LCB_331	11	25165	1154	TPM3+F+I+G4	-57315.1	0.1502
31	LCB_332	11	1324	78	K2P+I	-2898.84	0.1402
32	LCB_347	9	31227	901	HKY+F+G4	-64418.9	0.1096
33	LCB_351	10	2146	60	HKY+F	-4671.59	0.1304
34	LCB_357	10	23474	813	HKY+F+G4	-49042.2	0.1171
35	LCB_360	12	20203	976	HKY+F+I	-44137.8	0.138
36	LCB_363	12	7558	461	TPM3+F+G4	-18049.3	0.1879
37	LCB_364	11	3096	112	HKY+F+G4	-6320.24	0.106
38	LCB_366	9	30077	886	TPM2+F+G4	-62844.7	0.1152
39	LCB_367	11	2151	141	K3Pu+F+G4	-5343.64	0.2197
40	LCB_368	11	1609	67	HKY+F+G4	-3643.62	0.1555
41	LCB_381	10	25498	953	TPM2+F+I+G4	-57003.6	0.1509
42	LCB_385	10	3342	83	HKY+F+G4	-6808.16	0.1048
43	LCB_386	11	5497	248	HKY+F+G4	-12673.3	0.1626
44	LCB_387	11	13352	533	TPM3+F+G4	-29735.8	0.1412
45	LCB_390	12	8652	402	K3Pu+F+G4	-19048.3	0.132
46	LCB_391	12	2525	122	HKY+F+I	-5679.88	0.1478

47	LCB_400	10	2389	96	HKY+F+G4	-5504.88	0.1602
48	LCB_402	10	13925	394	HKY+F+G4	-29264.2	0.1201
49	LCB_417	10	5097	205	HKY+F+G4	-11579.5	0.1515
50	LCB_420	12	10690	781	HKY+F+G4	-27186.5	0.1973
51	LCB_421	12	2656	156	HKY+F+G4	-5968.73	0.1355
52	LCB_423	12	8678	464	HKY+F+G4	-19369.4	0.1427
53	LCB_424	12	4020	151	HKY+F+G4	-8466.89	0.1167
54	LCB_425	12	6210	449	HKY+F+G4	-15897.1	0.1983
55	LCB_445	10	5432	156	HKY+F+G4	-12307.3	0.1606
56	LCB_446	10	7912	211	HKY+F+I	-16165	0.1058
57	LCB_453	11	5198	412	HKY+F+G4	-12084.8	0.1628
58	LCB_474	12	10350	394	HKY+F+G4	-22130	0.1219
59	LCB_475	12	22624	1135	HKY+F+I+G4	-49692	0.1386
60	LCB_476	10	10736	291	HKY+F+G4	-22174	0.1174
61	LCB_477	11	1532	62	TPM3+F+G4	-3043.37	0.1103
62	LCB_478	12	3947	194	HKY+F+I	-8709.49	0.1402
63	LCB_481	12	5151	195	HKY+F+G4	-10698.6	0.1116
64	LCB_492	12	16296	908	HKY+F+G4	-34595.1	0.1208
65	LCB_496	12	9266	515	HKY+F+G4	-21148.3	0.1528
66	LCB_498	11	17599	755	HKY+F+R2	-40128	0.1575
67	LCB_500	12	5571	267	HKY+F+R2	-12397.6	0.1484
	All	12	636266	25844		-1397693.1125 (s.e. 1773.4217)	9.5769

Table S5. Phylogenetic input and results for the five SDR genes.

**Gene**	**sequences**	**length (bp)**	**inform -ative sites**	**best-fit model**	**optimal lnL**	**sister group**
GME	11	1217	25	K2P	-2062.14	Fchil_6_B2
RPP0W	34	4294	329	HKY+F+G4	-9384.58	Fchil_7_B2
glucan endo-1	21	1308	388	HKY+F+G4	-4096.01	FxaC_6_B1
purple acid phosphatase	7	496	5	JC	-1050.69	FxaC_6_B1
unchar. protein	6	1477	84	TN+F	-4197.98	Uncertain

Table S6. Concordance between mapped target capture marker locations on the GP33 assembly and the genetic linkage groups.

Female Chromosome	LG	Total markers	Markers from the same chromosome	Markers from a different homeolog of the same base chromosome	Markers from a different base chromosome
Fchil1-Av	8	50	48	2	0
Fchil1-B1	1	28	26	2	0
Fchil1-B2	3	24	22	2	0
Fchil1-Bi	4	26	24	2	0
Fchil2-Av	11	67	63	4	0
Fchil2-B1	5	47	44	3	0
Fchil2-B2	10	57	47	9	1
Fchil2-Bi	12	27	24	2	1
Fchil3-Av	17	51	43	8	0
Fchil3-B1	6	28	26	1	1
Fchil3-B2	23	28	27	1	0
Fchil3-Bi	16	22	19	3	0
Fchil4-Av	29	54	47	7	0
Fchil4-B1	27	34	32	2	0
Fchil4-B2	28	33	20	13	0
Fchil4-Bi	7	20	17	2	1
Fchil5-Av	33	59	58	1	0
Fchil5-B1	34	49	42	7	0
Fchil5-B2	30	31	26	4	1
Fchil5-Bi	9	40	32	7	1
Fchil6-Av	24	63	55	4	4
Fchil6-B1	19	49	42	6	1
Fchil6-B2	38	36	31	5	0
Fchil6-Bi	20	73	65	8	0
Fchil7-Av	2	47	45	1	1
Fchil7-B1	42	44	35	9	0
Fchil7-B2	40	36	33	3	0
Fchil7-Bi	41	35	32	3	0

Table S6. (continued)

Male Chromosome	LG	Total markers	Markers from the same chromosome	Markers from a different homeolog of the same base chromosome	Markers from a different base chromosome
Fchil1-Av	8	35	35	0	0
Fchil1-B1	1	20	20	0	0
Fchil1-B2	3	16	15	1	0
Fchil1-Bi	4	21	19	2	0
Fchil2-Av	11	50	49	1	0
Fchil2-B1	5	35	34	0	1
Fchil2-B2	10	36	28	6	2
Fchil2-Bi	12	26	26	0	0
Fchil3-Av	17	41	40	1	0
Fchil3-B1	6	24	24	0	0
Fchil3-B2	23	23	22	1	0
Fchil3-Bi	16	15	15	0	0
Fchil4-Av	29	38	37	1	0
Fchil4-B1	27	27	26	1	0
Fchil4-B2	28	31	20	11	0
Fchil4-Bi	7	14	14	0	0
Fchil5-Av	33	52	49	1	2
Fchil5-B1	34	28	26	2	0
Fchil5-B2	30	14	14	0	0
Fchil5-Bi	9	21	15	6	0
Fchil6-Av	24	79	79	0	0
Fchil6-B1	19	41	39	2	0
Fchil6-B2	38	45	45	0	0
Fchil6-Bi	20	41	36	3	2
Fchil7-Av	2	40	39	1	0
Fchil7-B1	42	17	17	0	0
Fchil7-B2	40	15	13	2	0
Fchil7-Bi	41	35	33	2	0

Table S7. Conserved single copy orthologs (BUSCOs) by subgenome and genome for the octoploids *F. chiloensis* GP33 and *F.* × *ananassa* 'Camarosa' and diploids *F. vesca* and *F. iinumae*. N=2326.

	**complete**	**single**	**duplicate**	**fragmented**	**missing**
GP33_Av	95.2%	92.7%	2.5%	1.0%	3.8%
GP33_B1	81.7%	79.5%	2.2%	2.1%	16.2%
GP33_B2	81.6%	78.7%	2.9%	1.8%	16.6%
GP33_Bi	86.8%	82.3%	4.5%	1.4%	11.8%
GP33_all	99.0%	3.1%	95.9%	0.2%	0.8%
Camarosa_Av	89.0%	77.9%	11.1%	1.0%	10.0%
Camarosa_B1	78.9%	75.6%	3.3%	1.8%	19.3%
Camarosa_B2	81.6%	76.2%	5.4%	2.5%	15.9%
Camarosa_Bi	88.9%	82.6%	6.3%	1.4%	9.7%
Camarosa_all	99.1%	3.7%	95.4%	0.1%	0.8%
vesca	98.2%	96.1%	2.1%	0.6%	1.2%
iinumae	97.4%	95.3%	2.1%	0.5%	2.1%

Table S8. Duplicated conserved single copy orthologs (BUSCOs) by chromosome in the octoploids *F. chiloensis* GP33 and *F.* × *ananassa* 'Camarosa' and diploids *F. vesca* and *F. iinumae*. Shaded counts are >4× the diploid chromosome.

**chromosome**	**GP33**	**Camarosa**	**chromosome**	**vesca**	**iinumae**
Av_1-4	12	15	1	14	10
Av_2-2	16	18	2	13	13
Av_3-4	18	17	3	12	19
Av_4-3	13	82	4	12	13
Av_5-1	23	91	5	16	13
Av_6-1	34	81	6	21	14
Av_7-2	11	224	7	16	12
subgenome total	127	528	Total	104	94
B1_1-1	17	34			
B1_2-1	28	24			
B1_3-1	12	26			
B1_4-1	18	15			
B1_5-4	14	11			
B1_6-2	20	36			
B1_7-4	10	24			
subgenome total	119	170			
B2_1-3	13	24			
B2_2-3	41	16			
B2_3-3	14	20			
B2_4-2	37	13			
B2_5-2	10	19			
B2_6-4	16	14			
B2_7-1	12	162			
subgenome total	143	268			
Bi_1-2	12	69			
Bi_2-4	18	19			
Bi_3-2	12	13			
Bi_4-4	129	17			
Bi_5-3	20	13			
Bi_6-3	26	127			
Bi_7-3	8	54			
subgenome total	225	312			

Table S9. Chromosomal location and size of telomeres. Bold indicates both telomeres were assembled.

GP33 chromosome	chromosome location	distance (bp) to chromosome end	length (bp)
Fchil1-Av	5'	16	1187
Fchil1-B1	5'	1	1190
Fchil1-B2	5'	1	1112
Fchil1-B2	interstitial	1646438	105
Fchil1-B2	interstitial	12252106	151
Fchil1-Bi	none		
**Fchil2-Av**	5' or interstitial	270018	1298
**Fchil2-Av**	3'	0	999
Fchil2-B1	5'	16879	1017
Fchil2-B1	interstitial	8712246	121
**Fchil2-B2**	5'	1	1207
**Fchil2-B2**	3'	2	1100
**Fchil2-Bi**	5'	1	1096
**Fchil2-Bi**	3'	0	1053
Fchil2-Bi	interstitial	15204595	103
Fchil3-Av	3'	1	863
Fchil3-B1	none		
Fchil3-B2	5'	1	1076
Fchil3-B2	interstitial	14457307	115
Fchil3-Bi	interstitial	24253127	104
Fchil3-Bi	interstitial	1886773	119
Fchil4-Av	3'	0	1072
Fchil4-B1	5'	1	1292
Fchil4-B2	3'	10539	1064
Fchil4-B2	interstitial	19792249	122
Fchil4-Bi	3'	15901	1201
Fchil4-Bi	interstitial	18568406	112
**Fchil5-Av**	5'	1	1313
**Fchil5-Av**	3' or interstitial	2434773	855
Fchil5-Av	interstitial	3913948	132
**Fchil5-B1**	5'	24055	768
**Fchil5-B1**	3'	0	1091
Fchil5-B1	interstitial	3808286	109
**Fchil5-B2**	5'	21444	1113
**Fchil5-B2**	3'	0	1219
Fchil5-Bi	3'	14816	1128
Fchil5-Bi	interstitial	3829478	105
Fchil6-Av	5'	1	852
Fchil6-B1	5'	16	1170

Fchil6-B2	5'	130	980
**Fchil6-Bi**	5'	11	1129
**Fchil6-Bi**	3'	30409	976
Fchil6-Bi	interstitial	27772226	102
**Fchil7-Av**	5'	1	1257
**Fchil7-Av**	3'	0	1093
**Fchil7-B1**	5'	1	1339
**Fchil7-B1**	3'	0	1123
Fchil7-B2	3'	0	1199
Fchil7-Bi	3'	0	1065

Table S10. Regions of homeologous exchange (HE) in the W haplotig alignment. The SDR is located between *F. vesca* chromosome 6 position 38499517-38499527. The closest regions of HE are marked with an asterisk and are 169 kbp and 406 kbp away.

alignment block	length (bp)	homeologous exchange	vesca chrom 6 start	vesca chrom 6 end	combined length (bp)
LCB_268	24403	HE Fvb6-2	38091470	38115872	
LCB_267	16836	HE Fvb6-2	38121270	38138105	
combined	41239		38091470	38138105	46635
LCB_321	9496	HE Fvb6-2	38315210	38324707	
LCB_320	3828	HE Fvb6-2	38324708	38328537	
LCB_332	2318	HE Fvb6-2	38328538	38330857*	
combined	15642		38315210	38330857	15647
LCB_476	11878	HE Fvb6-2	38905957*	38917834	
LCB_300	18232	HE Fvb6-2	38967100	38985331	
LCB_351	3137	HE Fvb6-2	38985332	38988468	
LCB_357	25208	HE Fvb6-2	38988469	39013676	
combined	58455		38905957	39013676	107719
LCB_150	16562	HE Fvb6-3	39414823	39431384	16561

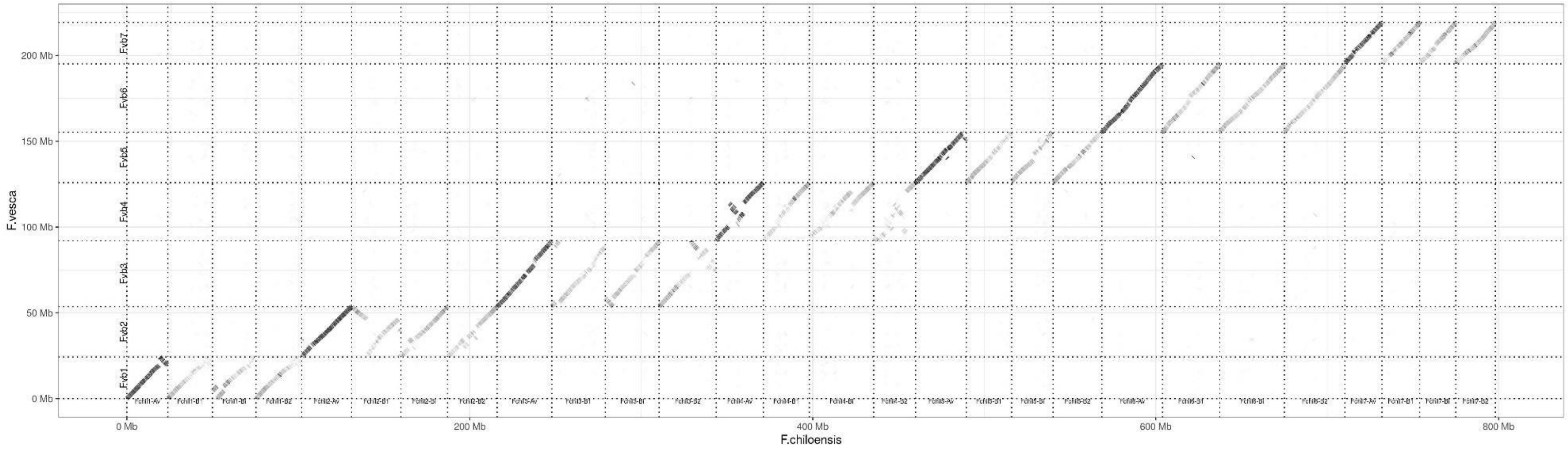

Fig. S1: Dotplot of a minimap2 alignment between F. chiloensis (octoploid) assembly and F. vesca (diploid).

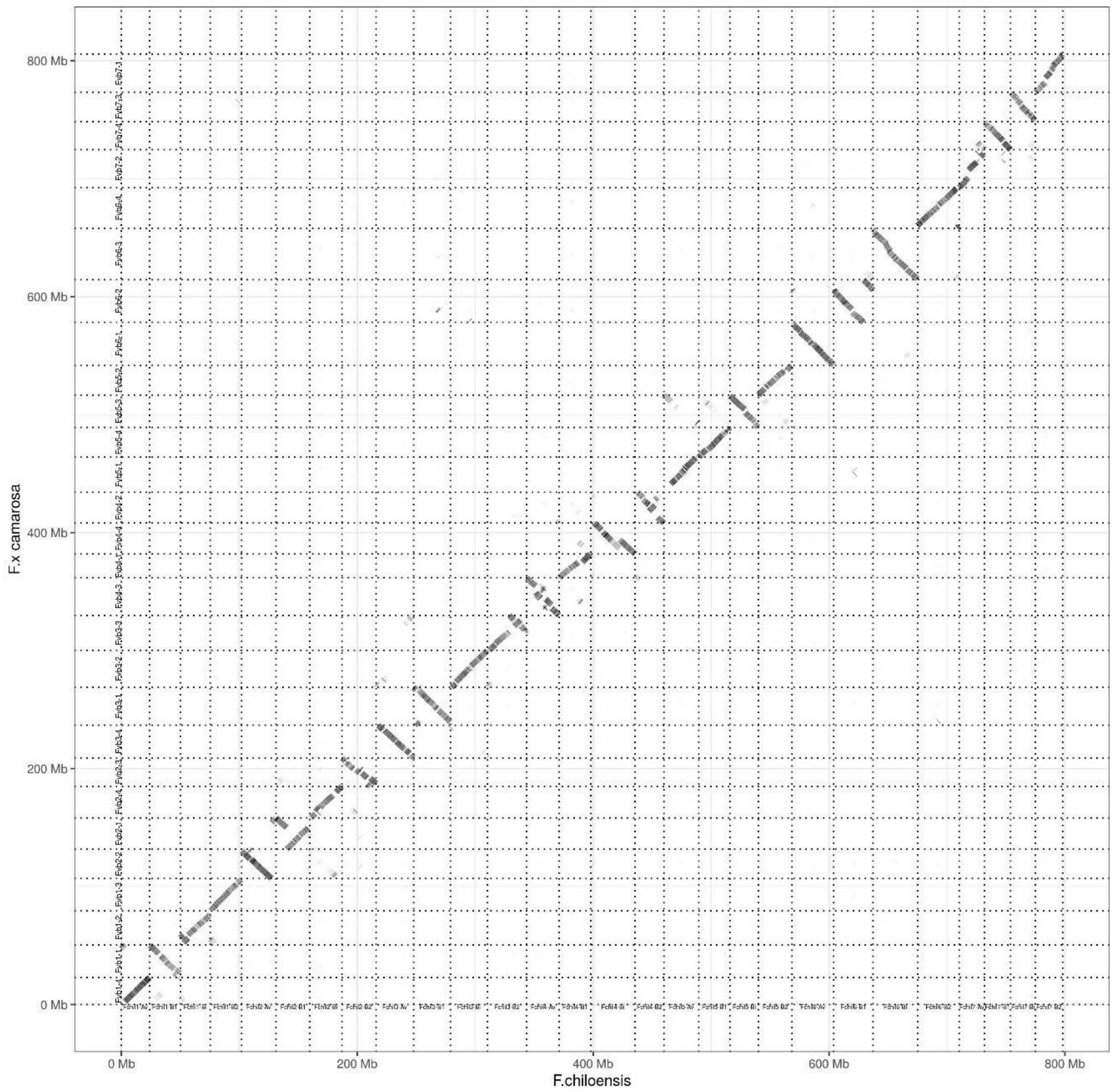

Fig. S2:Dotplot of a minimap2 alignment between F. chiloensis (octoploid) assembly and F. x 'Camarosa' (octoploid).

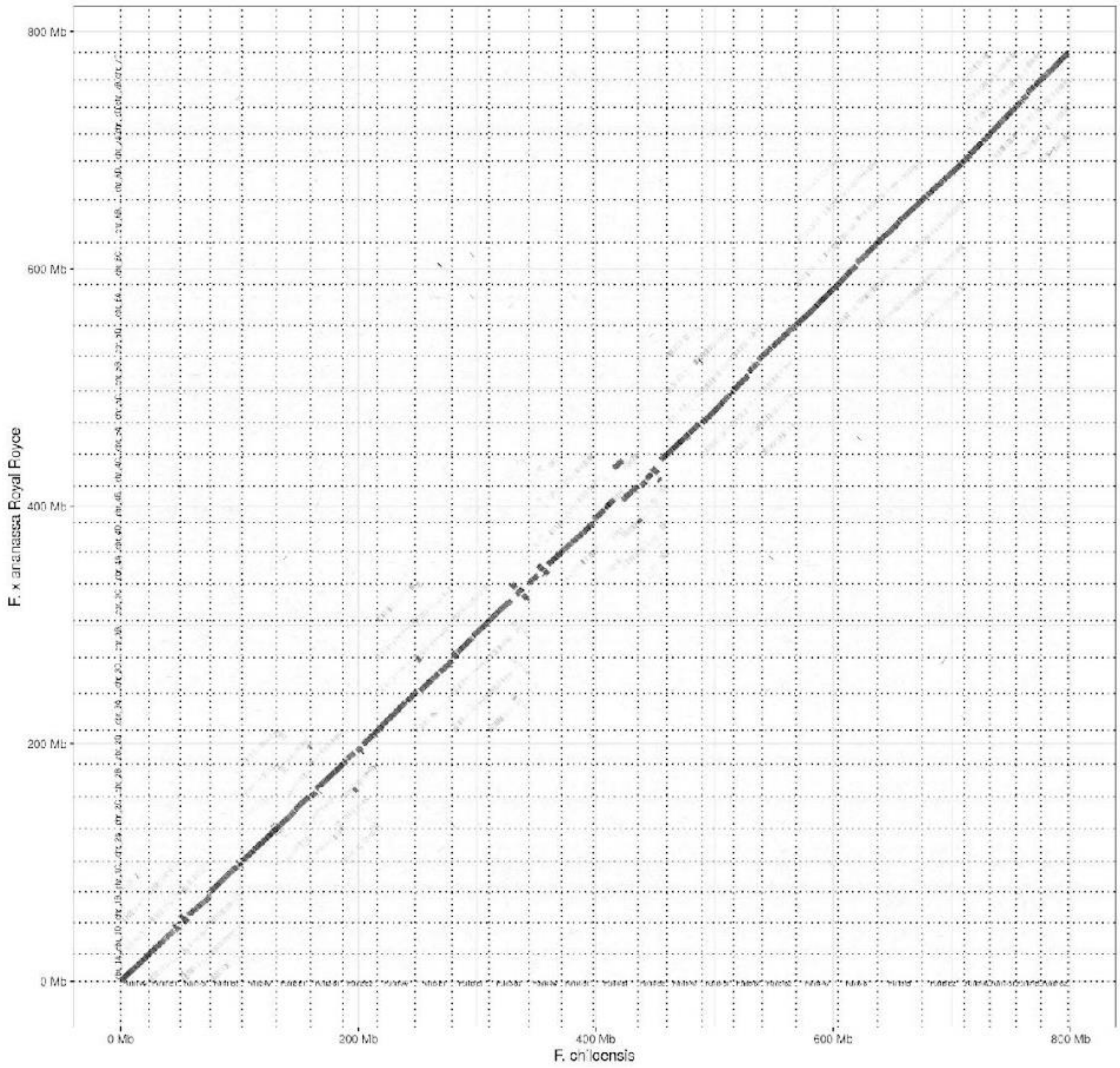

Fig. S3: Dotplot of a minimap2 alignment between F. chiloensis (octoploid) assembly and F. x 'Royal Royce' (octoploid).

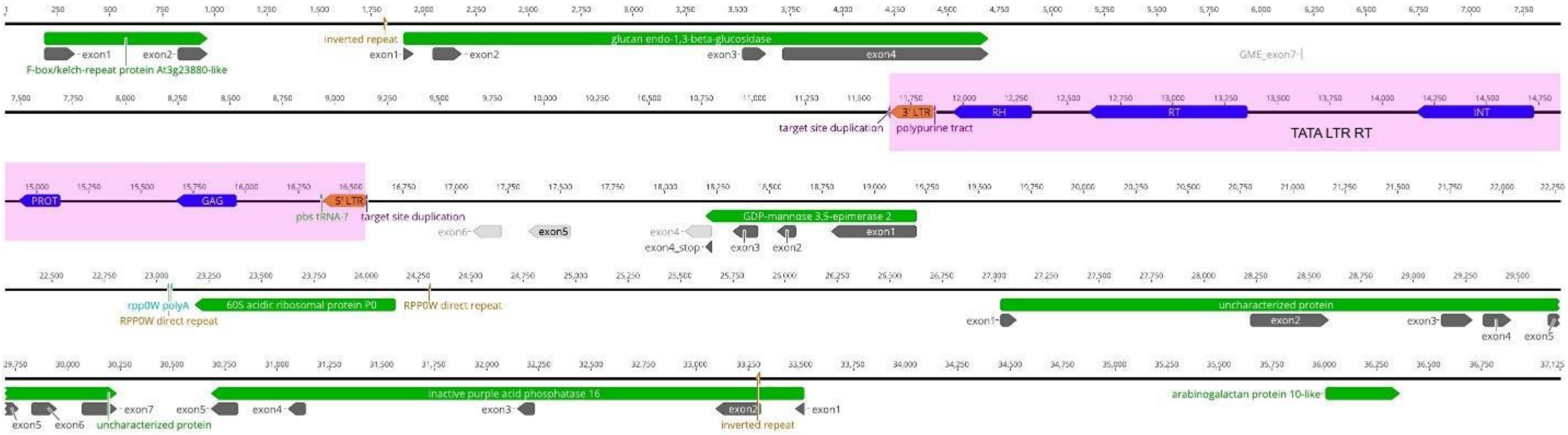

Fig. S4: Diagram of the W SDR with gene and LTR retrotransposon annotation. The non-canonical (TATA) LTR RT is shaded in pink. GDP-mannose 3',5' epimerase (GMEW) has a premature stop codon relative to the ancestral sequence - the original exons 4-7 are shaded in light gray.

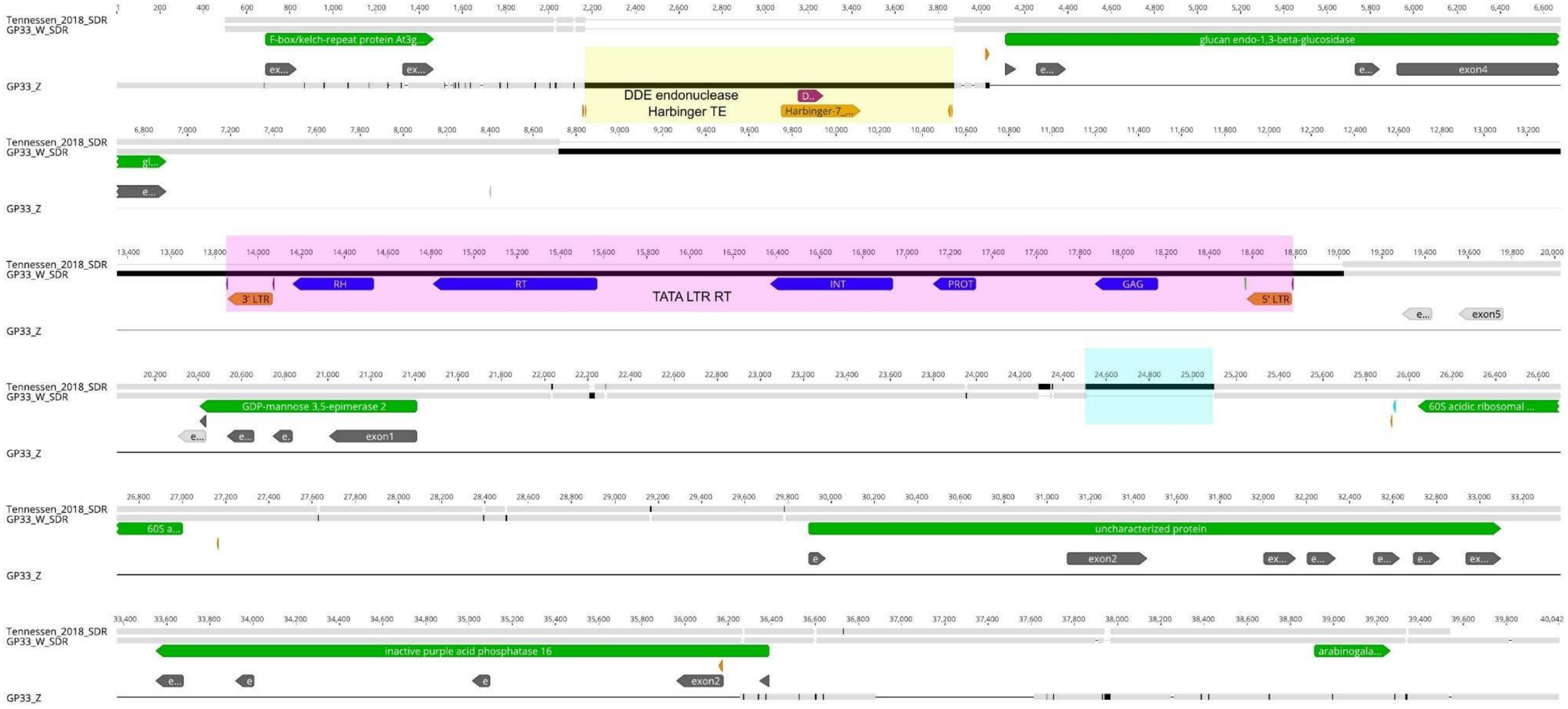

Fig. S5: Diagram of the MAFFT alignment of the W SDR (previously published and current versions) and homologous Z sequence. The Z-specific Harbinger TE is shaded in yellow. The non-canonical (TATA) LTR RT is shaded in pink. The originally misassembled hAT TE is shown in light blue.

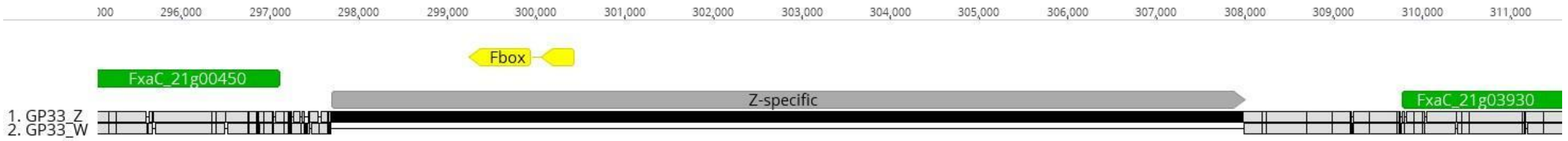

Fig. S6: Diagram of a subset of an AnchorWave alignment between the Z and W sequence of GP33. The Z specific region with no W homolog is highlighted in grey. The predicted coding region of the Z specific Fbox gene is in yellow. Genes with homologs on the Z and W annotated with the liftoff program are in green. The Figure was obtained from Geneious R9.1.

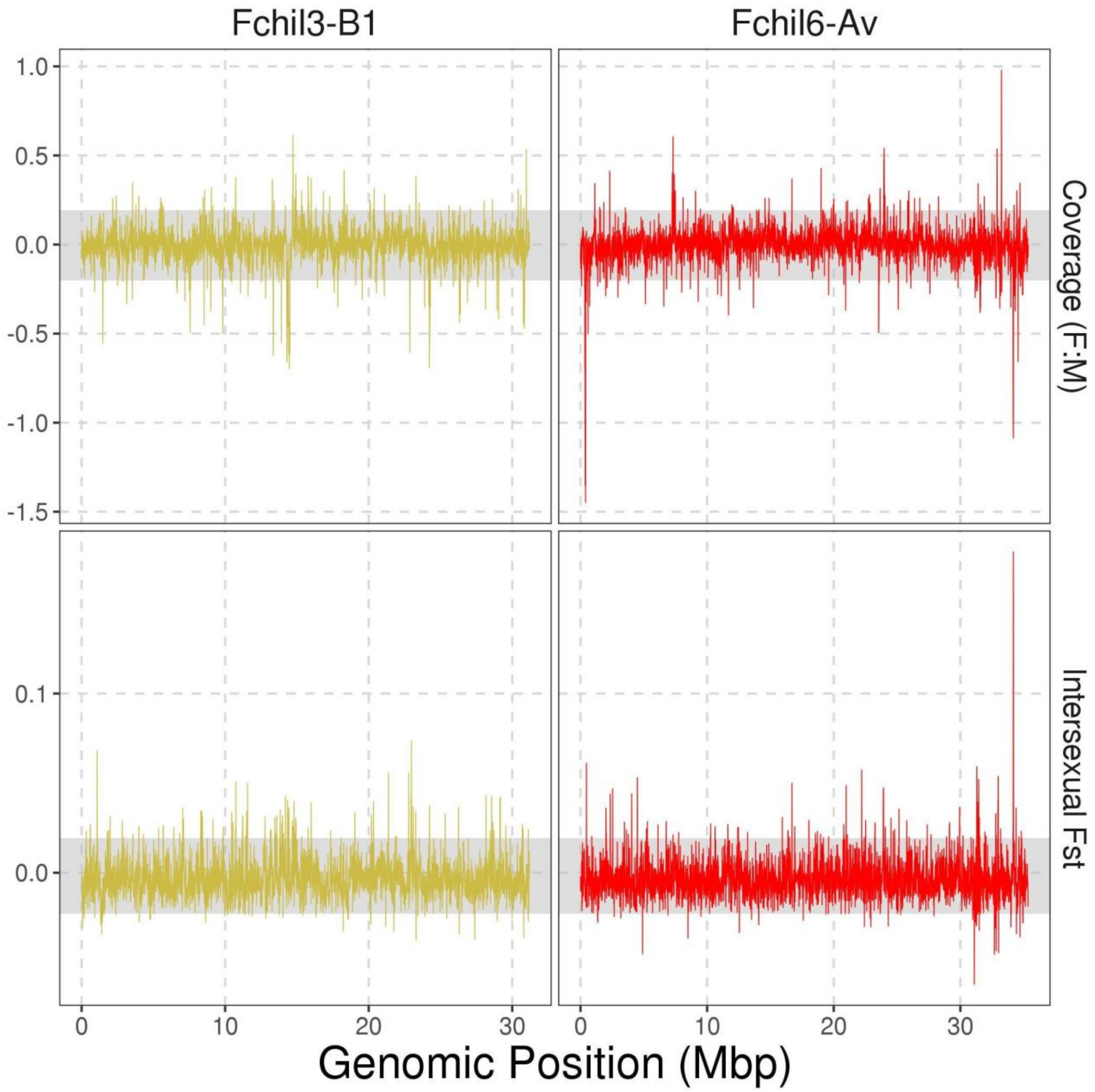

Fig. S7: Comparison of the sex chromosome Fchil6-Av and an autosome Fchil3-B1 for male vs. female coverage (log2(mean F) - log2(mean M)) and intersexual weighted Fst in non-overlapping 10kb windows. The grey area represents the 95% confidence interval obtained by resampling a representative autosome (Fchil3-B1) 1000 times.

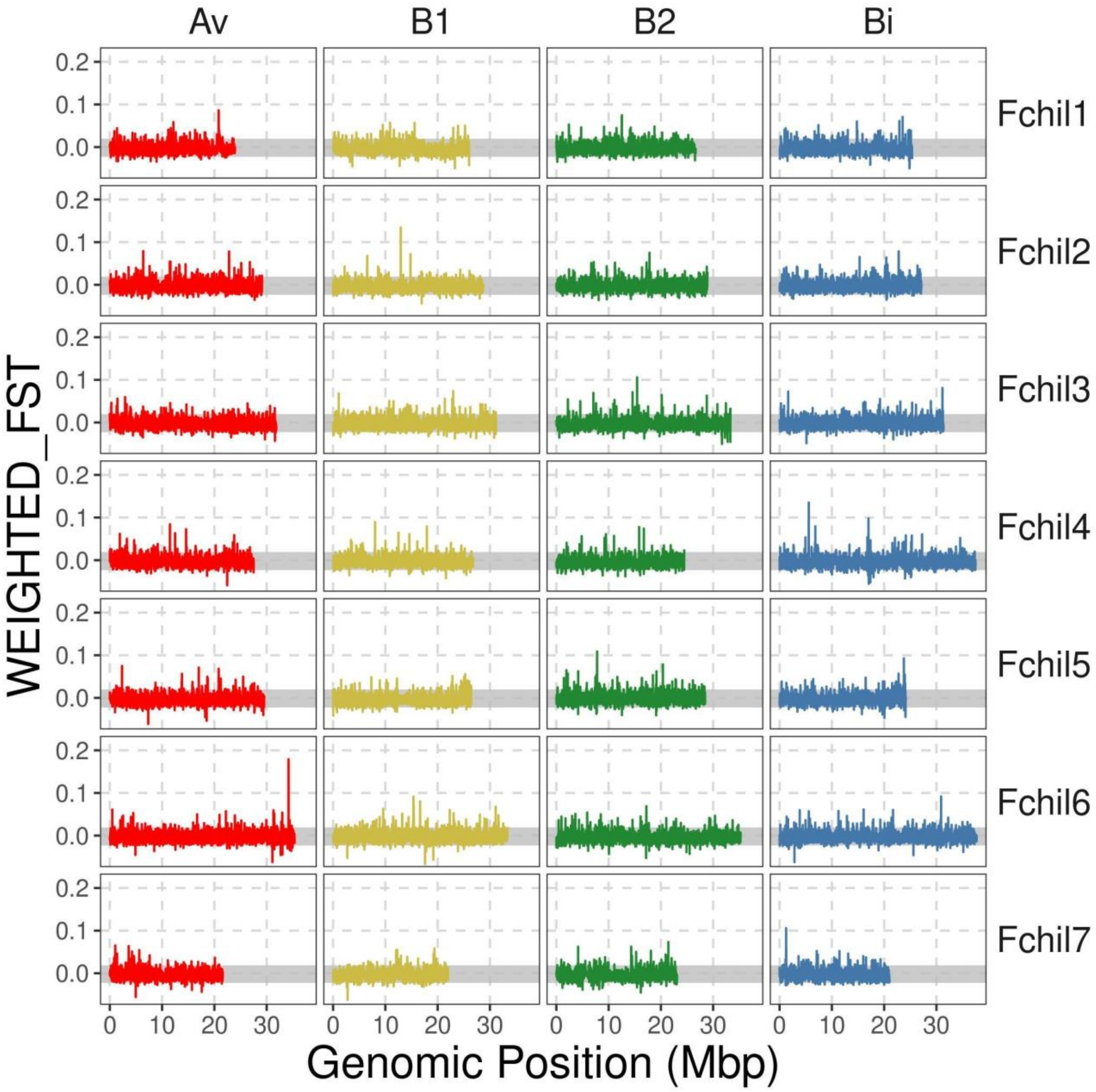

Fig. S8: Intersexual weighted Fst on non-overlapping 10kb windows across all 28 GP33 chromosomes. The grey area represents the 95% confidence interval obtained by resampling Fst values from a representative autosome (Fchil3-B1) 1000 times.

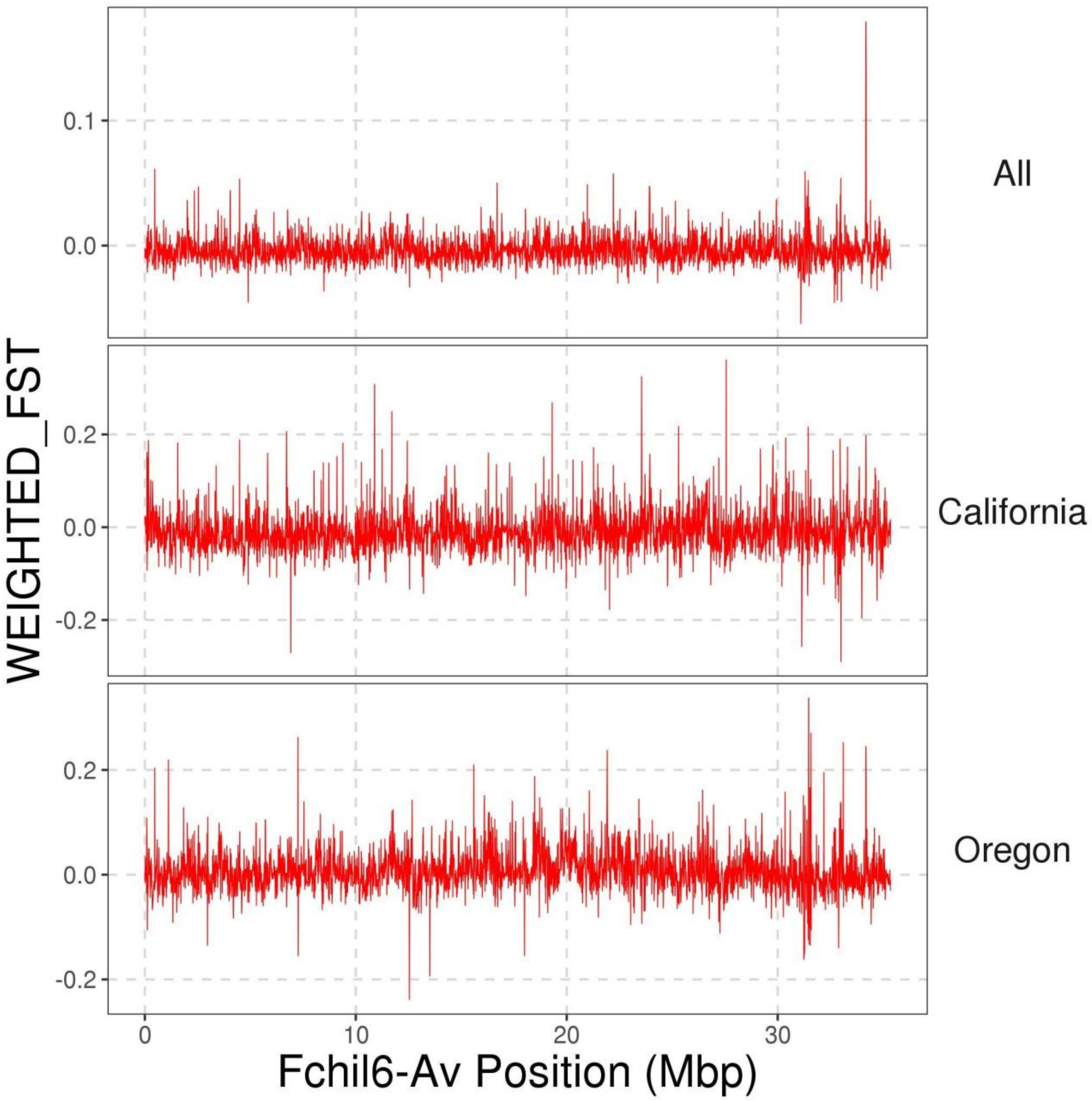

Fig. S9:Intersexual weighted Fst on non-overlapping 10kb windows on the sex chromosome Fchil6-Av. Comparison of all samples, California only, and Oregon only.

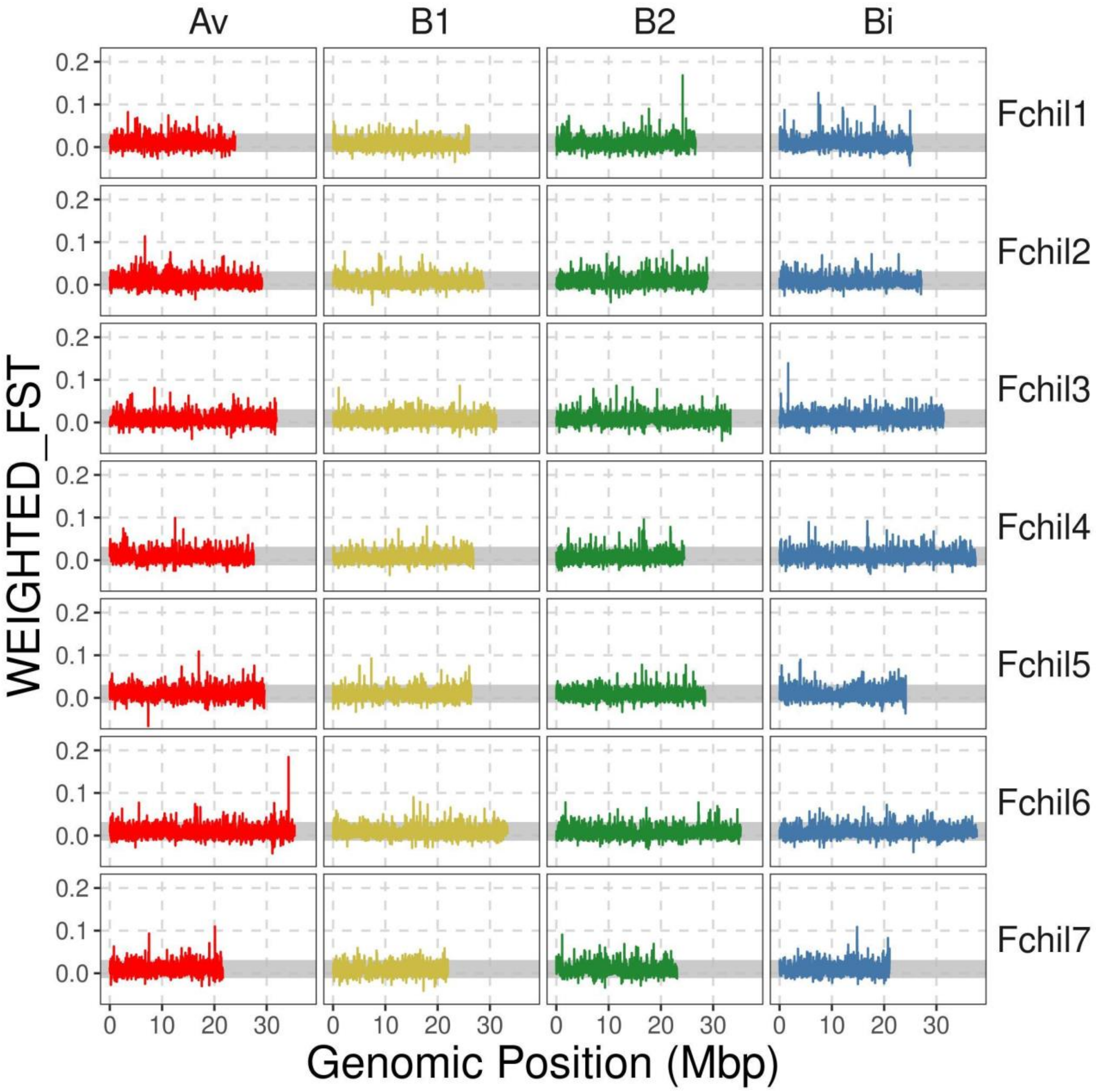

Fig. S10: Intersexual weighted Fst on non-overlapping 10kb windows across all 28 GP33 chromosomes for the full dataset (13 females and 25 males). The grey area represents the 95% confidence interval obtained by resampling Fst values from a representative autosome (Fchil3-B1) 1000 times.

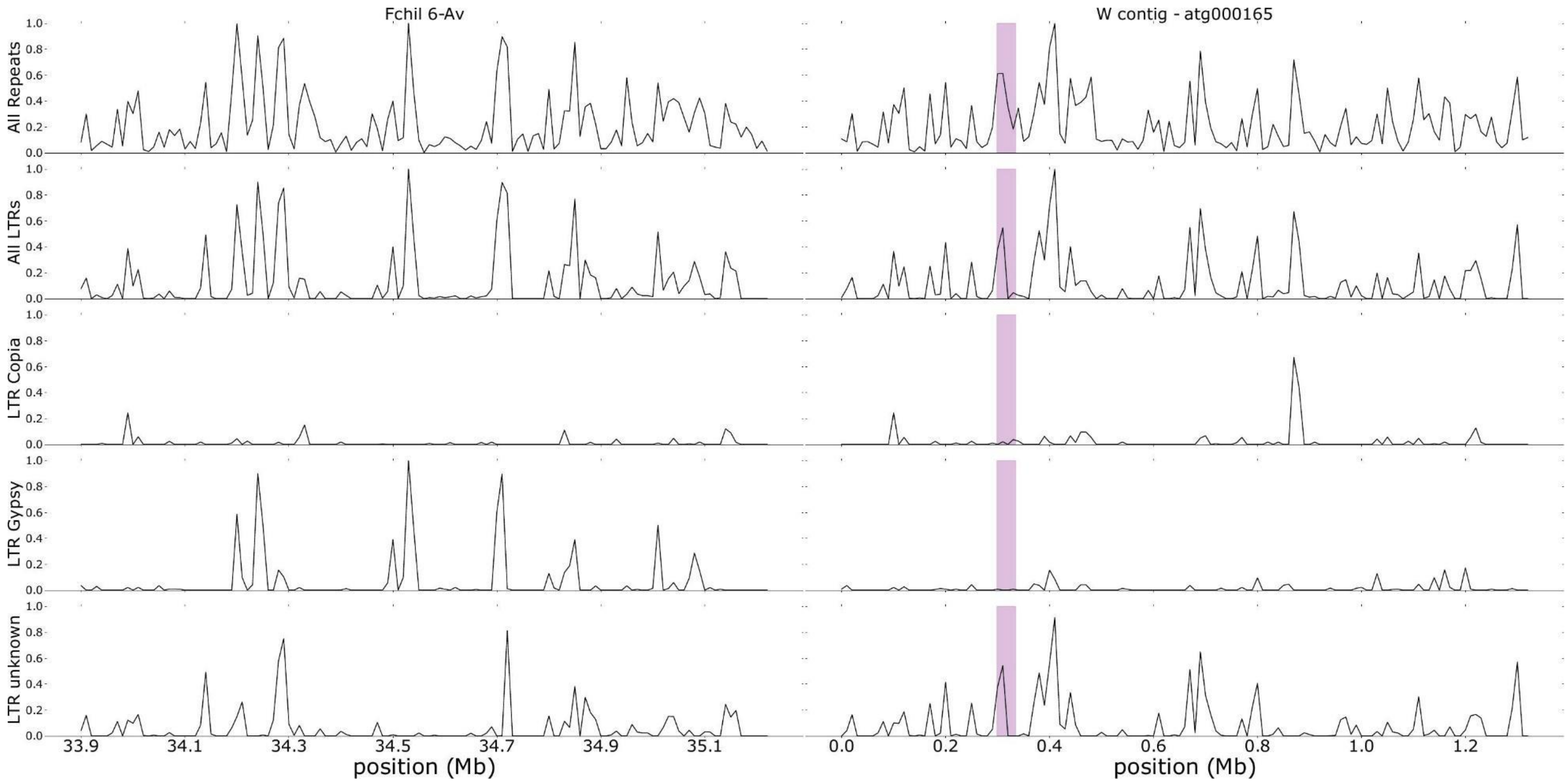

Fig. S11: Comparison of the abundance of all repeats and LTR RT superfamilies on the W haplotig and homologous sequence of the Z chromosome. The purple area represents the location of the SDR.

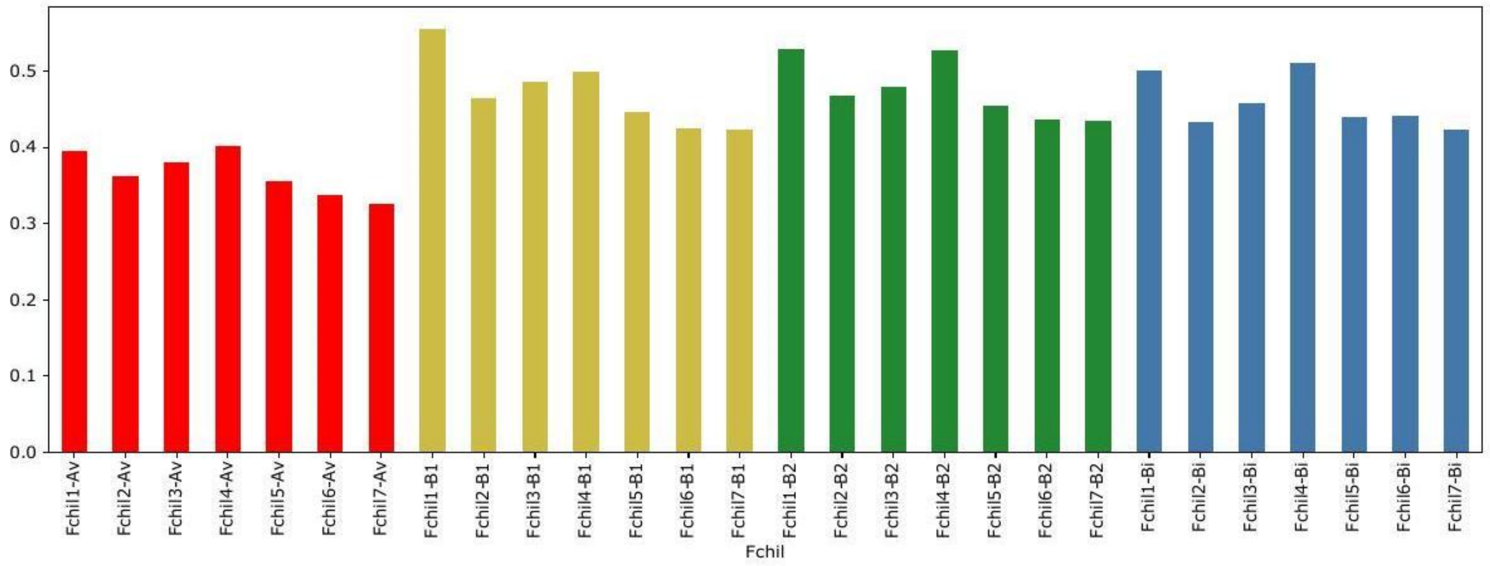

Fig. S12: Repeat coverage across the chromosomes of F. chiloensis. The colors correspond to the subgenome they originated from: Av (red), B1 (yellow), B2 (green), and Bi (blue).

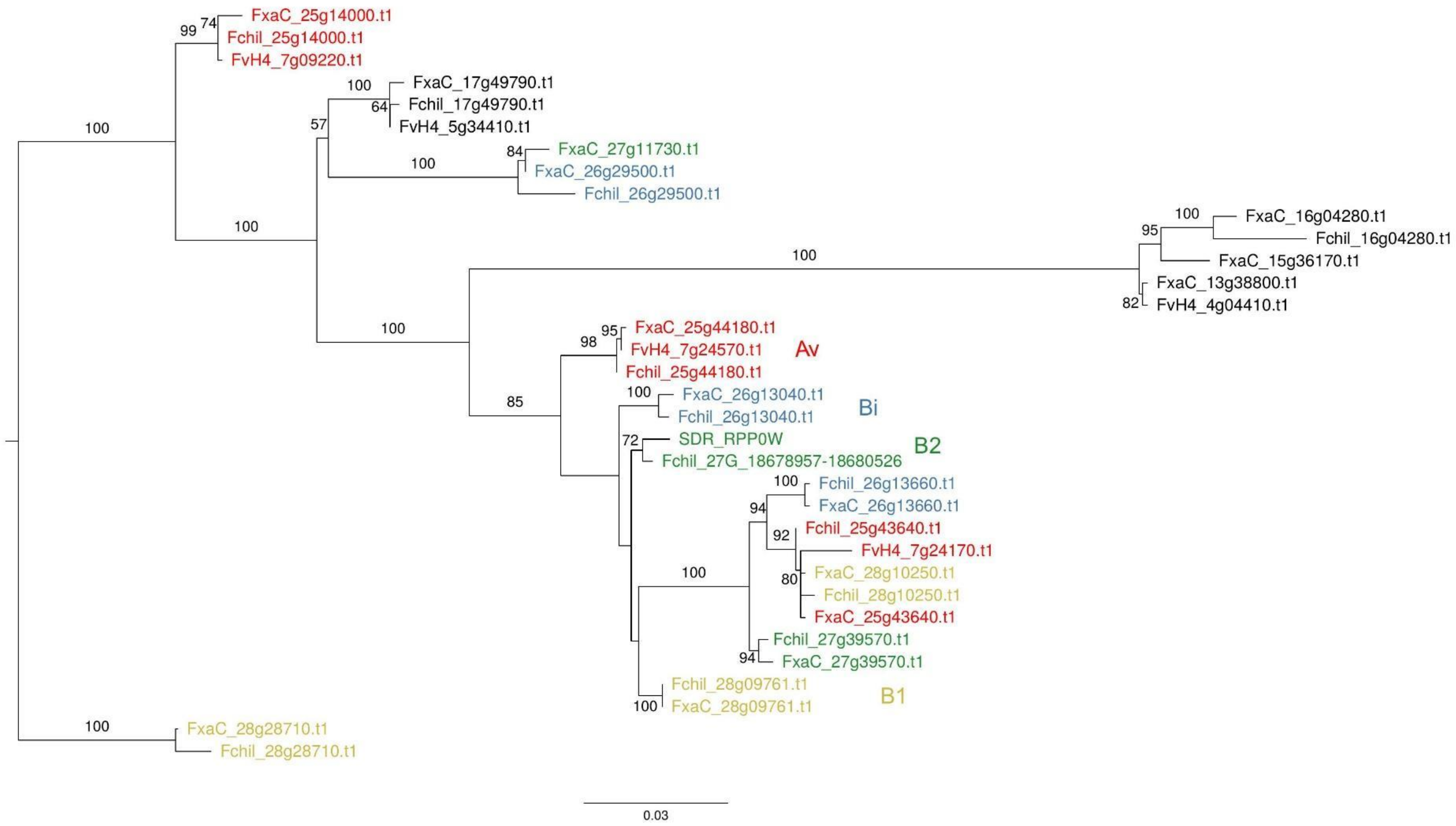

Fig. S13: Maximum likelihood phylogenetic tree for ribosomal protein P0 (RPP0) with bootstrap values. For gene prefixes, Fchil is Fragaria chiloensis, FxaC is F. x ananassa 'Camarosa', and FvH4 is F. vesca. Colors correspond to the subgenome of origin: Av (red), B1 (yellow), B2 (green), and Bi (blue).

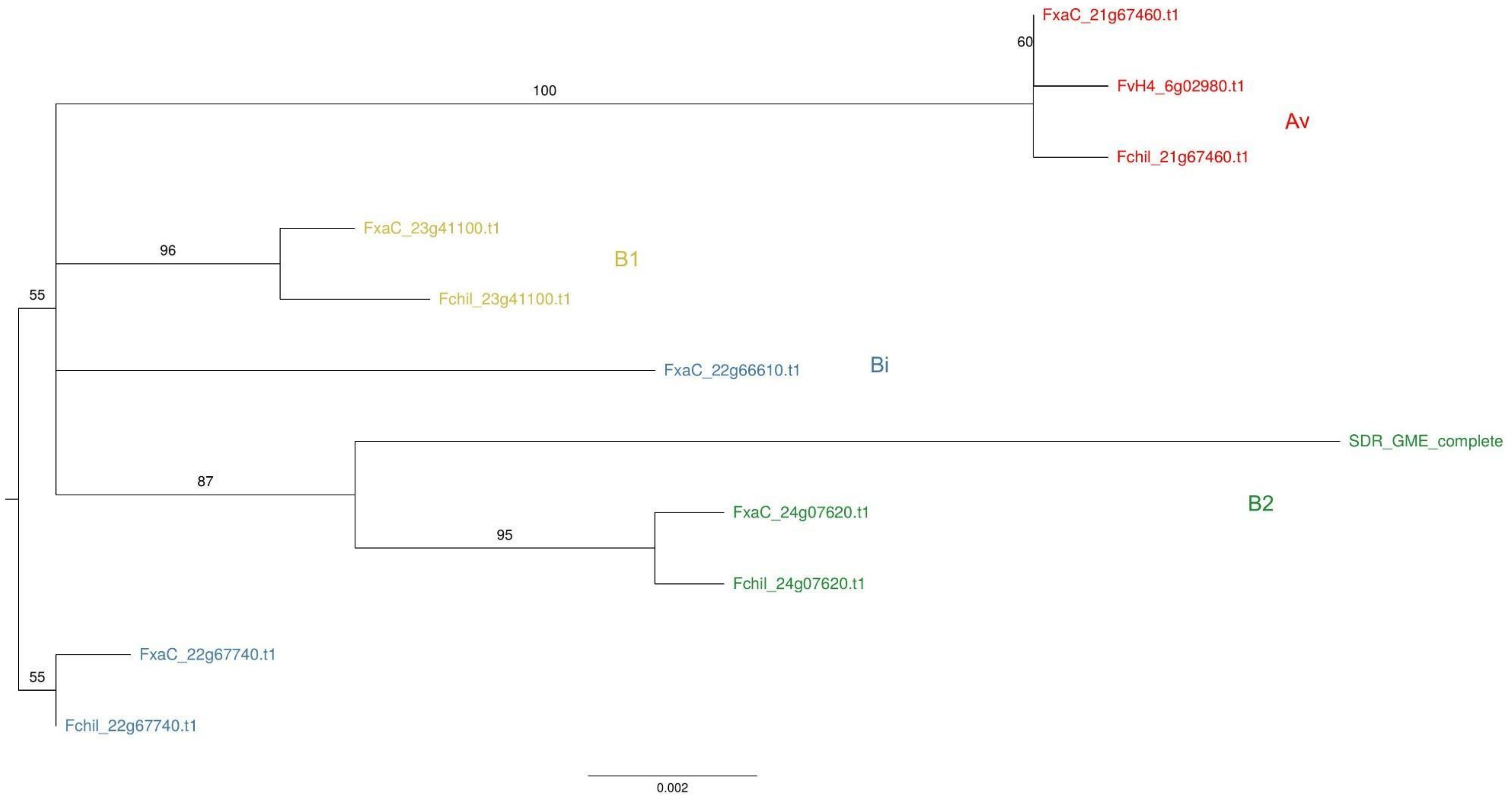

Fig. S14: Maximum likelihood phylogenetic tree for GDP-mannose-3',5'-epimerase (GME) with bootstrap values. For gene prefixes, Fchil is Fragaria chiloensis, FxaC is F. x ananassa 'Camarosa', and FvH4 is F. vesca. Colors correspond to the subgenome of origin: Av, B1 (yellow), B2 (green), and Bi (blue).

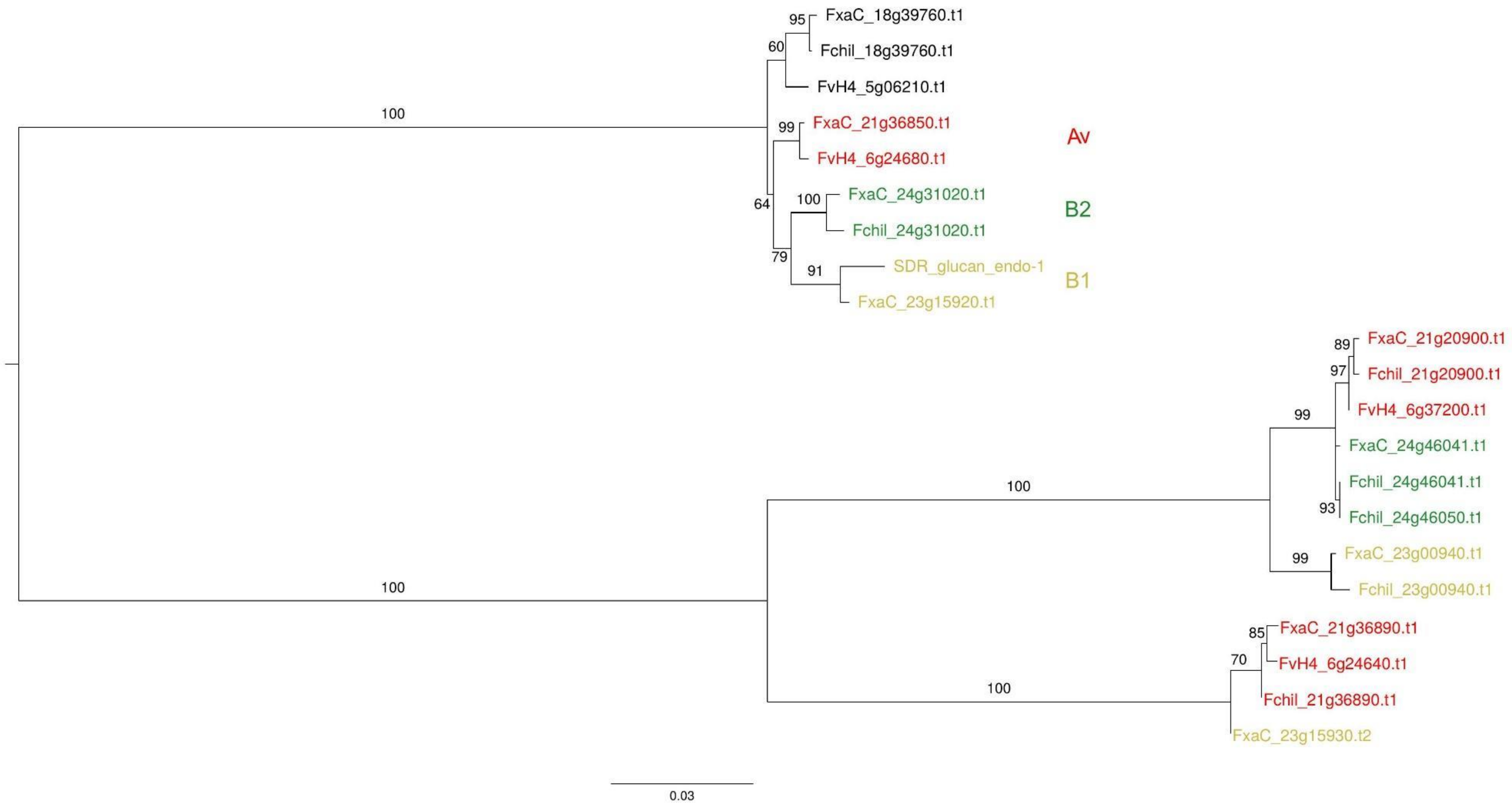

Fig. S15: Maximum likelihood phylogenetic tree for glucan endo-1,3 beta-glucosidase with bootstrap values. For gene prefixes, Fchil is Fragaria chiloensis, FxaC is F. x ananassa 'Camarosa', and FvH4 is F. vesca. Colors correspond to the subgenome of origin: Av (red), B1 (yellow), B2 (green), and Bi (blue).

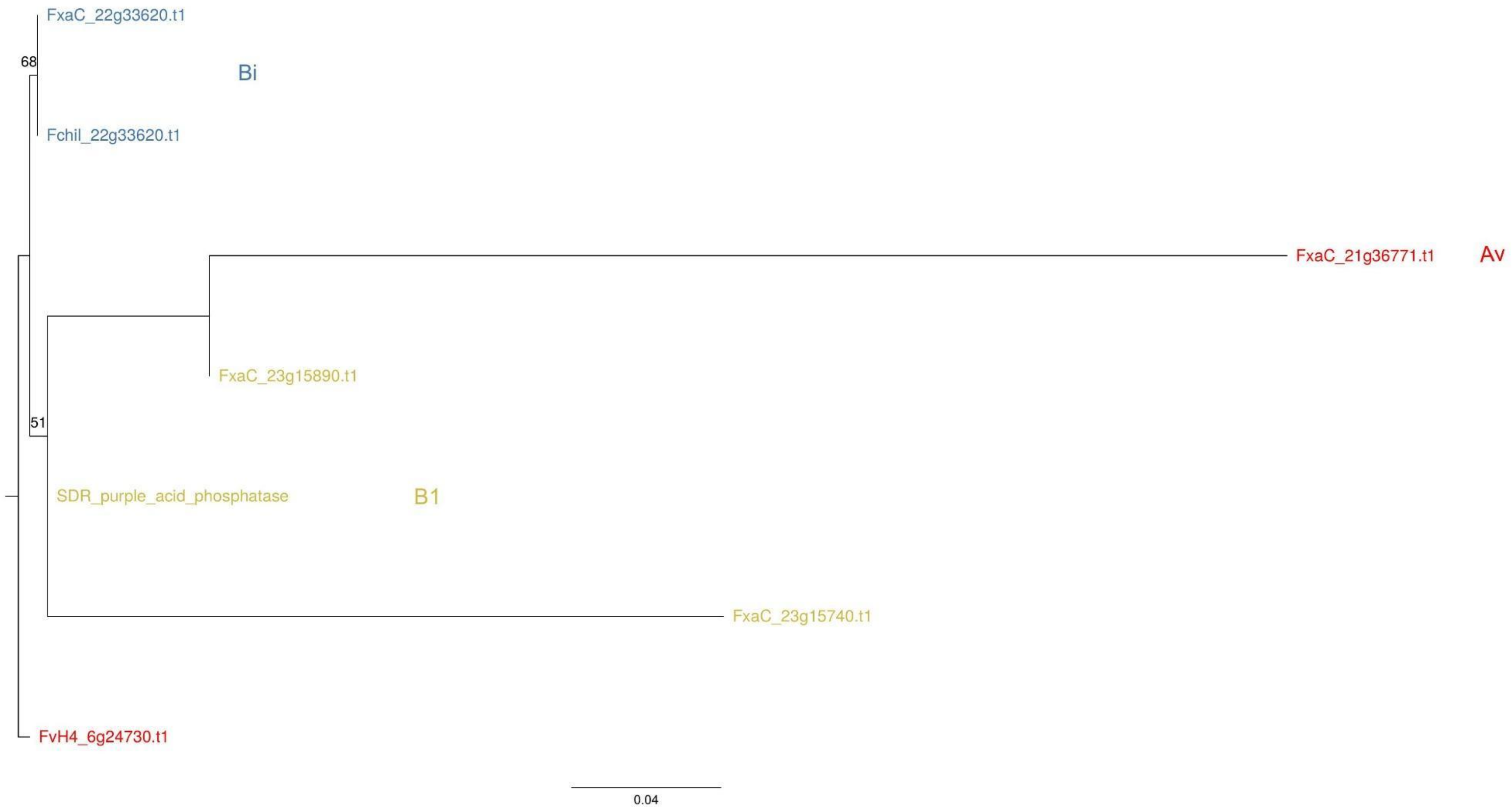

Fig. S16: Maximum likelihood phylogenetic tree for inactive purple acid phosphatase 16 with bootstrap values. For gene prefixes, Fchil is Fragaria chiloensis, FxaC is F. x ananassa 'Camarosa', and FvH4 is F. vesca. Colors correspond to the subgenome of origin: Av (red), B1 (yellow), B2 (green), and Bi (blue).

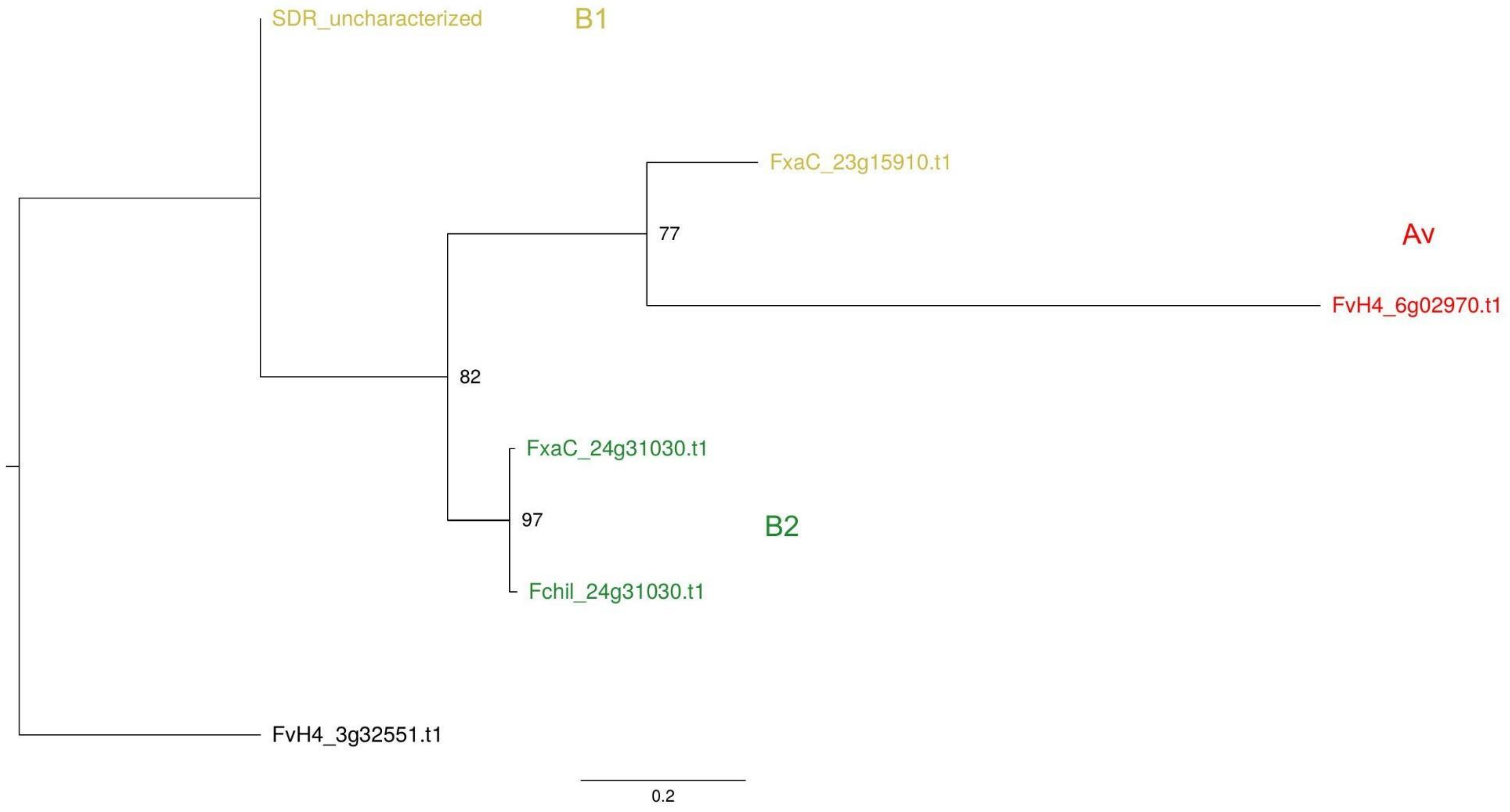

Fig. S17: Maximum likelihood phylogenetic tree for the SDR uncharacterized protein with bootstrap values. For gene prefixes, Fchil is Fragaria chiloensis, FxaC is F. x ananassa 'Camarosa', and FvH4 is F. vesca. Colors correspond to the subgenome of origin: Av (red), B1 (yellow), B2 (green), and Bi (blue).

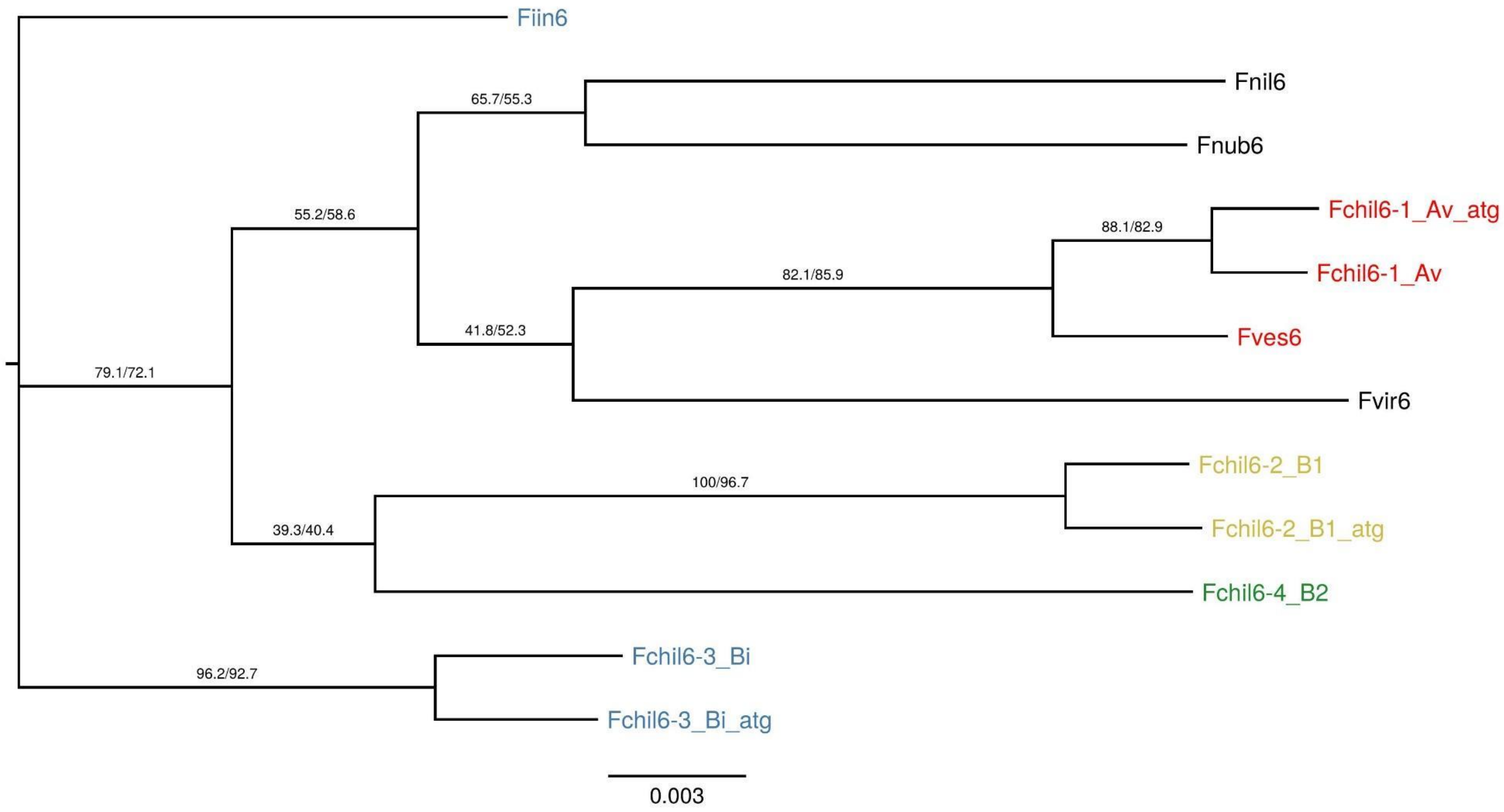

Fig. S18: Maximum likelihood phylogenetic tree for the haplotype-resolved Z and W, the other chromosome 6 homeologs (autosomes) and diploid Fragaria species. For gene prefixes, Fchil is Fragaria chiloensis, FxaC is F. x ananassa 'Camarosa', and FvH4 is F. vesca. Colors correspond to the octoploid subgenome and diploid progenitor: Av (red), B1 (yellow), B2 (green), and Bi (blue).
